# Influence of phase-separated structural morphologies on the piezo and triboelectric properties of polymer composites

**DOI:** 10.1186/s11671-023-03868-8

**Published:** 2023-07-01

**Authors:** Swathi Yempally, Eya Kacem, Deepalekshmi Ponnamma

**Affiliations:** 1 Center for Advanced Materials, Qatar University, P O Box 2713, Doha, Qatar; 2 Materials Science and Technology Program, Department of Mathematics, Statistics and Physics, College of Arts and Sciences, Qatar University, 2713 Doha, Qatar

**Keywords:** Nanogenerators, Phase separation, Frictional, Mechanical, Deformation

## Abstract

Simplified and flexible fabrication methods, high output performance, and extreme flexibility of polymer-based nanocomposites represent versatile designs in self-powering devices for wearable electronics, sensors, and smart societies. Examples include polyvinylidene fluoride and its copolymers-based piezoelectric nanogenerators, green and recyclable triboelectric nanogenerators, etc. Advanced functionalities, multi-functional properties, and the extensive lifetime required for nanogenerators inspire researchers to focus on structural modifications of the polymeric materials, to fully exploit their performances. Phase separation is a physicochemical process in which polymeric phases rearrange, resulting in specific structures and properties, that ultimately influence mechanical, electronic, and other functional properties. This article will study the phase separation strategies used to modify the polymeric base, both physically and chemically, to generate the maximum electric power upon mechanical and frictional deformation. The effect of interfacial modification on the efficiency of the nanogenerators, chemical and mechanical stability, structural integrity, durable performance, and morphological appearance will be extensively covered in this review. Moreover, piezo- and triboelectric power generation have numerous challenges, such as poor resistance to mechanical deformation, reduced cyclic performance stability, and a high cost of production. These often depend on the method of developing the nanogenerators, and phase separation provides a unique advantage in reducing them. The current review provides a one-stop solution to understand and disseminate the phase separation process, types and mechanisms, advantages, and role in improving the piezoelectric and triboelectric performances of the nanogenerators.

## Introduction and background survey

Wearable and portable electronics, wireless transport systems, and Internet of things (IoT) technology are demanding sustainable power generation methodologies [1–3]. Smart societies, including cloud computing and artificial intelligence, utilize such energy generators and sensors for real-time monitoring of remote or short-range data [2, 3]. Wearable electronic technology realizes intelligence and acts as the best interface for human–machine interaction, but it needs proper power supplies for constant and stable performance [4]. The miniaturization of electronic devices, portability, and wearable healthcare monitoring systems gained significance in research after the year 2000 [5–7]. More than 15,000 research papers dealing with wearable electronics and around 2000 publications for the year 2022 underline the significance of these topics. Mechanical power harvesting methods such as piezo- and tribotools often connect with wearable electronics and smart society for continuous power supply [8, 9]. Moreover, these power generation strategies are renewable and do not involve any environmental concerns.

Piezoelectric nanogenerators, or PENGs, generate electrical energy from the difference in surface potential of a crystalline material with non-central symmetry when an external mechanical force deforms it [10], whereas the triboelectric nanogenerators, or TENGs, generate electrical energy by coupling triboelectrification and electrostatic induction in materials [4]. Extensive studies demonstrate the piezoelectric power generation performances of piezoelectric ceramics [11], inorganic oxides (ZnO [12, 13], BaTiO_3_ [14], SrTiO_3_ [15], and tellurium [16]), hybrid composites, and organic–inorganic hybrid materials (surface modification by sol–gel reaction, chemical bonding, electrostatic adsorption, and nanocoating) [17, 18]. In order to understand the mechanism of piezoelectricity in polymer nanocomposites and the various materials used for fabricating PENGs, the review of Mishra et al. [19] is helpful. Our research group has done extensive research on the piezoelectric properties of polyvinylidene fluoride (PVDF) [20, 21] and its copolymer, polyvinylidene fluoride hexafluoropropylene (PVDF-HFP) [22, 23]. Depending on the synthesis method and the nature of the nanofillers used, the piezoelectric nanocomposites showed different rates of performance. For instance, a maximum piezoelectric output voltage of 12 V and a current density of 1.9 µA cm^−2^ for the PVDF-HFP/cellulose nanocrystal (2 wt%) and PVDF-HFP/Fe-doped ZnO (2 wt%) double-layer composite spun fibers were 60 times higher when compared to the neat polymer [12]. However, with Co-doped ZnO of the same concentration, single-layer fibers of PVDF-HFP only produced 2.8 V [24]. At the same time, for silver nanoparticle-loaded PVDF spun fibers, an output voltage in the range of 0.6–2 V was observed when the concentration changed from 0.2 to 0.4% [20]. On the other hand, the voltage was relatively low when fabrication methods other than electrospinning were used. Though ZnO was doped with nickel before embedding in the PVDF-HFP, the layer-by-layer deposition method generated a maximum voltage of only 1.2 V that too at a 0.5 wt% nanomaterial concentration [25]. It was also observed that *γ*-irradiation significantly improved the *β*-phase nucleation and thus the piezoelectric output voltage for PVDF films [12]. In this case, the crystallinity was 48.1% for the casted films of PVDF containing Fe-doped ZnO, with a maximum output voltage of 2.4 V. It is clear from all these reports that the crystallinity and *β*-phase nucleation are greatly influenced by the morphological deformations happening within the polymer because of the nature and type of nanofiller, their concentration, type of fabrication, and processing conditions.

Similarly, studies on TENGs are also advancing, with variable output performance coming from the multilayer structure, fabrication methods, and post-fabrication modifications. A very recent study by Choi and co-workers [26] reports polydimethylsiloxane (PDMS)-based carbon black (CB) composites as TENGs, demonstrating excellent charge storage ability, design flexibility, and material selection diversity. Optimized CB content gave twice the enhancement in long-term output performance with an output current of 320 μA. Ferroelectric composite interfacial layers are developed by Cao et al. [27] by doping nylon with polymethyl methacrylate (positive triboelectricity) and by compositing PVDF-TrFE with ZrO_2_ nanoparticles (negative triboelectricity). After polarizing in a specific direction, a high frictional surface charge density of 220 μC/m^2^ was achieved with respective open-circuit voltage, output power density, and short-circuit current density of ~ 500 V, ~ 42 mW cm^−2^ and ~ 500 μA cm^−2^. A recent review article published by Walden’s research group [28] focuses on the various types of TENGs and their different working mechanisms, including separation and sliding modes of operation. They classified the TENGs based on their triboelectric nature as tribonegative polymer material-based (PTFE, fluorinated ethylene propylene, PVDF, PDMS, polyimide, PET, and grafted polymeric layers) and tribopositive polymer material-based (nylon, cellulose, silk, thermoplastic polyurethane, polyvinyl alcohol, melamine formaldehyde, peptides, etc.). This review highlighted the significance of structural and morphological features in triboelectric power generation, such as tilting-sensitive TENGs [29], pendulum-inspired TENGs [30], whirling-folded TENGs [31], bionic stretchable TENGs [32], ball-shell-structured TENGs [33], and lawn-structured TENGs [34]. It is well understood that the structural topographies influence the electric power generation in all triboelectric power generation systems [29] and so polymers are always modified to attain specific structural and morphological identities.

Structural induction of nanomaterials and non-solvents to align the –CH_2_ and –CF_2_ units in PVDF and thus enhance the efficiency of PENG was done during the phase separation process [34]. The phase separation happens according to the ternary phase diagram and Flory–Huggins [35] theory, by which at a point of demixing, evaporation, and condensation due to the solvent and non-solvent of the polymer will induce the phases to separate. In addition to incorporating phase separation as an additional method for electrospinning [35], the process can be coupled with the normal casting method as well. Depending on the nature and mechanism, phase separation process is classified in to several types, such as vapor induced, non-solvent induced, and thermal induced. 3D porous PVDF-HFP/Fe_3_O_4_ nanocomposites were fabricated by Shen’s research group using the scalable and template-free, non-solvent-induced phase separation (NIPS) method [36]. They could achieve highly durable piezoelectric power generation with a power density of 5.3 μW/cm^3^ and a piezoelectric *d*_33_ coefficient of 48.6 pC/N by modulating nanoparticle migration and liquid–liquid mixing. Yan and co-workers [4] used the same process of phase separation in a coagulation bath (NIPS) as a post-fabrication method to develop tribo-electronegative and tribo-electropositive pairs using PTFE/PVDF/EVOH composites for a possible smart garment. With a power density of 2.45 W/m^2^, the developed TENG harvests frictional energy between the clothes triggered by human motions. A very recent study [37] demonstrated the phase separation nanocoating method of covering the BaTiO_3_ with PVDF-TrFE particles, in which the 1-octanol non-solvent induces the phase separation. Such coated BaTiO_3_ was thereafter casted into the polymer films to develop TENGs with a high output voltage of 59.5 V. The vapor-induced phase separation (VIPS) process to manufacture self-matched configurations of PENG/TENG using recombinant spider silk and PET/PVDF reports another innovative concept of inducing higher triboelectric output from PENG’s piezoelectric effect [38]. Phase separation helps in the dipole alignment within the polymer chains, which adds to the enhanced energy harvesting properties. In addition, recent reports also discuss the advantages of phase separation of fluorine-rich polymer, poly(2,3,4,5,6-pentafluorostyrene) (PPFS), from sulfur copolymer TENGs [39], concave-honeycomb GO-PLA with convex-PDMS phase-separated antagonistic friction surfaces [40] for bio-TENGs, and sausage-like strings of PVDF nanodomains [41].

Based on all these studies, it is well established that phase separation acts as an alternative method to generate or modify polymer nanocomposites for both piezo- and triboelectric power generation. In this review, we will collectively assemble all the phase separation strategies used for PENG and TENG development, emphasizing their mechanism, role in morphological changes, and contribution to characteristic performance. The correlative aspects of the phase separation process on the structural variations and alignment of various phases will be discussed in detail, along with the nature of nanofillers, concentration, and other processing parameters. A critical discussion of the published articles on PENGs and TENGs as well as the combined nanogenerator will help the reader understand the phase separation process in polymers and its tuning in modulating specific properties. An effort like this is rarely found in the literature, and thus, we believe the scope of this review is relevant in recent times.

## Phase separation—types and mechanism

**Fig. 1:**
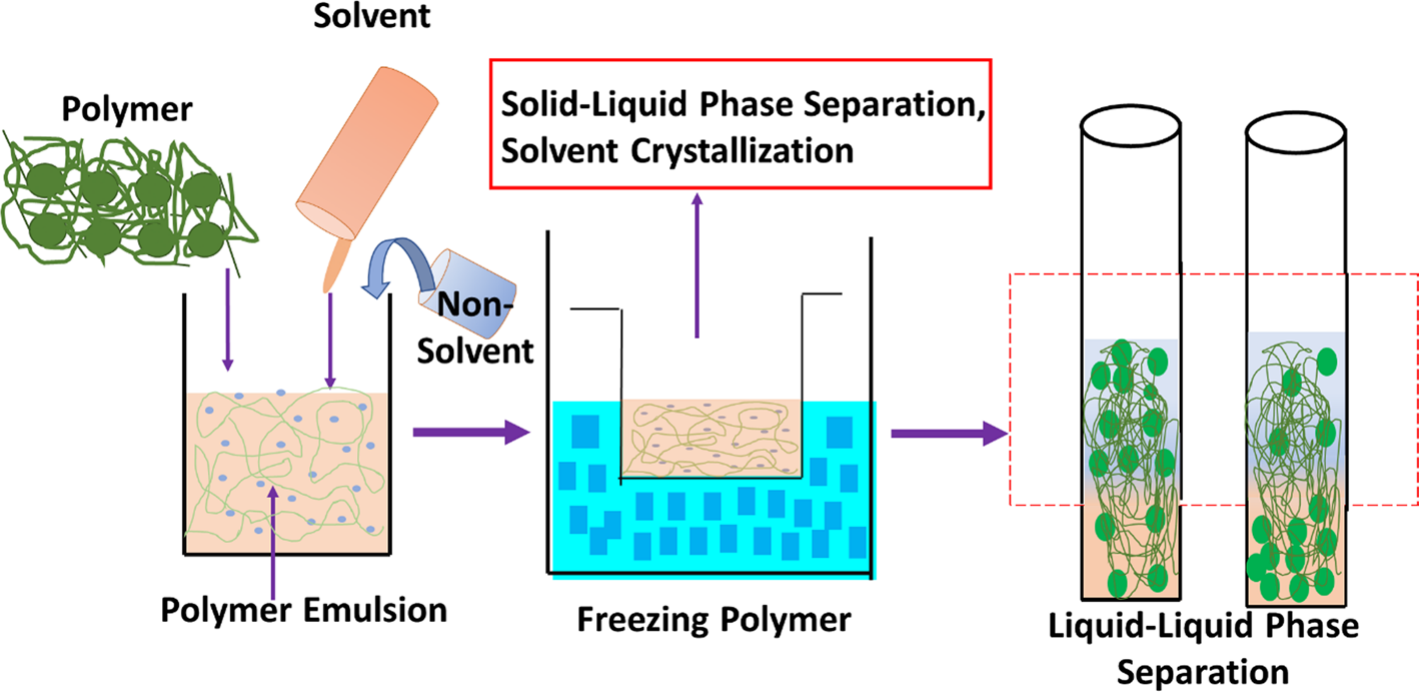
Schematic representation of a typical phase separation process

Phase separation occurs as two or more distinct phase formations in a single homogenous mixture when solubility parameters are affected. It is a type of phase transition in which molecules that have a tendency to adhere together split off into different compartments, territories, or bodies [42]. In polymers (both natural and synthetic), the phase separation process is initiated by dissolution in a suitable solvent to produce a polymer gel and thereafter evaporating it by typical solvent extraction strategies. One of the phases goes with the solvent, leaving the other rich polymeric phase, mainly due to physical incompatibility. The concentrations of molecules that tend to adhere together influence the phase separation. The driving forces for phase separation and the durations of non-covalent cross-links that develop among molecules are determined by the intensities of contacts and the number (valence) of contacting points. The combined impacts of networks of connections among molecules with the required high valence of contact points will aid in the organization of these molecules into discrete regions marked by a physical contact with the surrounding milieu—the phase boundary. Figure 1 represents the general phase separation process happening in a typical polymer system. Phase separation has much significance in different applications; for instance, in scaffold fabrication, phase separation regulates the shape by tuning the concentration, porogen type, polymer type, and freezing temperature. More clearly, biomedical applications such as tissue engineering needs well-defined pore structure and diameters. Phase separation can tune the porous structure formation, by changing the concentration of the polymer solution, type of the polymer, etc. In addition, the conditions involved in the phase separation methods like temperature (heating or freezing), solvent/non-solvent ratio, vapor pressure, etc.

**Fig. 2:**
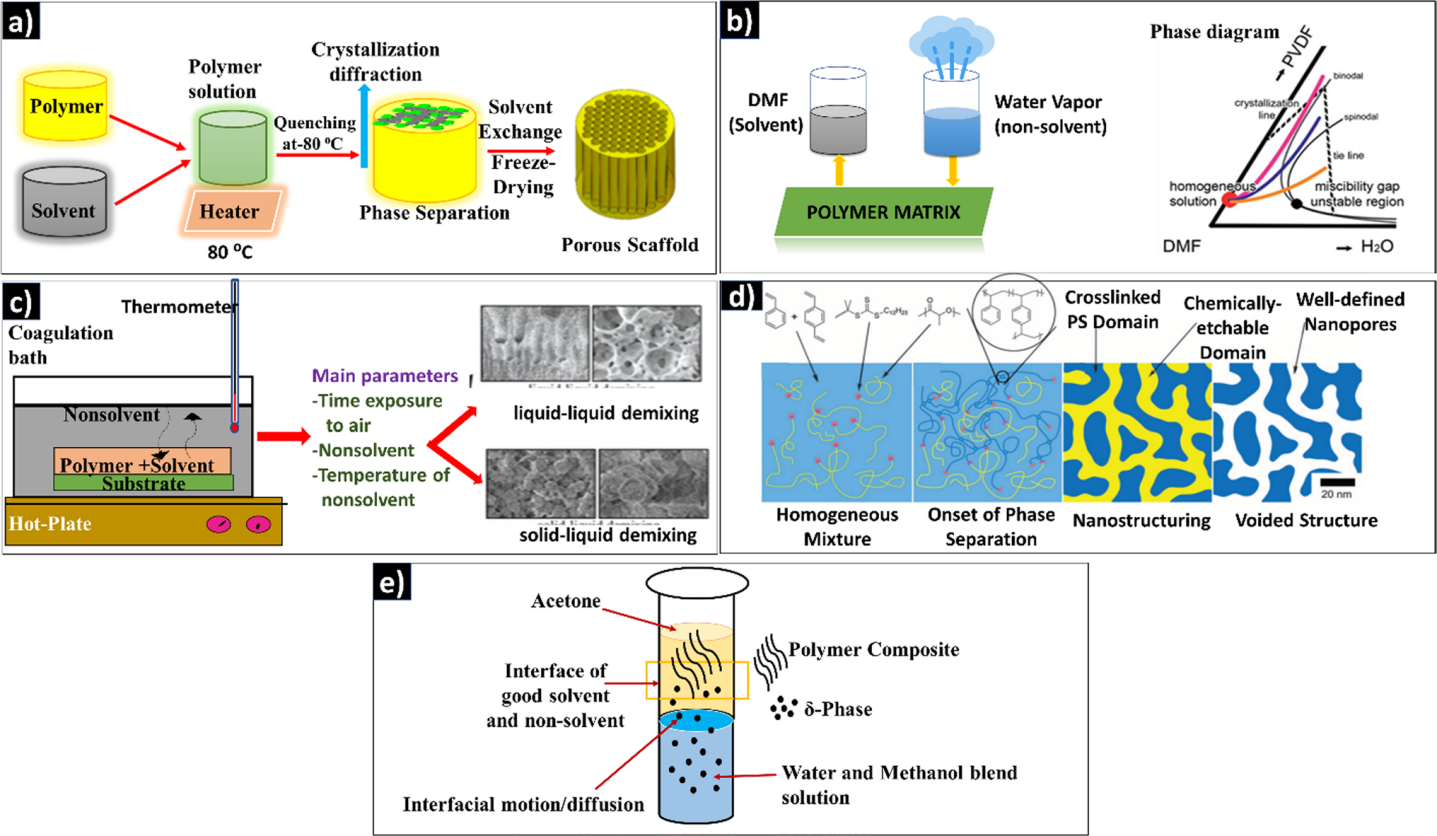
Types of phase separation. **a** Thermal-induced phase separation (TIPS); **b** VIPS and PVDF/[BMIM]BF_4_ phase diagram [44]. Licensed under a Creative Commons Attribution-Non Commercial 3.0 Unported License; **c** NIPS, types and parameters influencing [42]; **d** Morphologies derived from PIPS. Etchable polymer (yellow) dissolved in a monomer/cross-linker (light blue) medium and RAFT copolymerization generates block polymer structure with controlled chain growth (dark blue), expanding chains are additionally cross-linked in situ, Microphase separation and cross-linking stopping the emerging bi-continuous structure, and Removal of etchable polymer, resulting in percolating nanopores [45]; **e** Bi-solvent-induced phase separation (BIPS)

Specifically, the crystalline structure of PVDF-based polymers can be explained based on different electrochemical phases present in them, such as *α*-phase, *β*-phase, *γ*-phase, and *δ*-phase. The *α*-phase consists of PVDF chains in trans-gauche twist conformation (TGTG′), whereas the *β*-phase contains all-trans planar zigzag conformation (TTTT). The *γ*-phase and *δ*-phase, respectively, include (TTTGTTTG′) intermediate *α*- and *β*-conformation and a polar form of *α*-phase. Out of all these phases, the most common observed are *α* and *β*. Since the *α*-phase has a trans-gauche conformation (TGTG'), it cannot be polarized when subjected to an electric field, whereas in the all-trans *β*-phase (TTTT), the majority of fluorine atoms are separated from hydrogen atoms and thus have a dipole moment perpendicular to the polymer chain. The third type of chain orientation is the *γ*-phase, which is a transitional structure between the *α* and *β* phases and thus has a smaller dipole moment than the *β*-phase. This is due to its structure, which adopts a trans-gauche conformation with a higher trans fraction (TTTGTTTG'), whereas *β* and *γ* phases are polarizable. Interestingly, the piezoelectric property of PVDF and its copolymers mainly depends on the *β*-phase (as it is uniform in alignment); however, the monomer orientation is expensive and needs either mechanical stretching or high-voltage application [43]. In PVDF, different kinds of phase separation happen depending on the amount and nature of the phases present. Figure 2 schematically demonstrates five different kinds of phase separation processes happening within the PVDF-based polymers, such as thermal-induced, non-solvent-induced, vapor-induced, polymerization-induced, and bi-solvent-induced processes. The subsequent sections demonstrate the scope and mechanism of each process explaining the scheme (Fig. 2) in detail.

### Thermal-induced phase separation (TIPS)

In general, the TIPS relies on temperature changes, and [46] the crucial step is the dissolution of the polymer in the solvent with a high boiling point above the crystallization temperature [47], as represented in Fig. 2a. It has the following stages [48].

1. Mixing the low molecular weight polymer in a high boiling point liquid (diluent or latent solvent) to form a homogenous solution. (Liquid does not cause significant dissolution or swelling of the polymer at room temperature.)
2. Pouring the heated polymer solution into specific molds and shaping on a cold surface.
3. Solidification of polymers induces phase separation.
4. Generating the microporous structure by removing the diluent trapped in the polymer matrix during phase separation and solidification (by solvent extraction).
5. Post-treatment processes such as stretching improve the desired separation properties of the TIPS membranes.

When the phase separation happens highly porous scaffold structure can be developed. The advantages of TIPS include low defect formation, a wide range of solvent selection, and higher reproducibility [49]. However, the process can be monitored for uniform heating, and thermal stability of polymers.

### Vapor-induced phase separation process (VIPS)

VIPS uses water vapor as the non-solvent phase to induce phase separation by mixing with polymer solution and through condensation [50]. The phase diagram of VIPS included in Fig. 2b illustrates DMF, a high boiling solvent, as an example. During the polymer processing, water vapor from humidified air, completely miscible with DMF, diffuses into the film. Since DMF evaporates at a slower rate than water, PVDF phase separation occurs in the presence of non-solvent water [44]. The mechanism of VIPS can be either nucleation and growth [51] (metastable region) or spinodal decomposition (SD) [52] (unstable region), depending upon the location of phase separation composition on the thermodynamic phase diagram.

Several studies investigated the impacts of dissolving temperature (*T*_d_), vapor temperature (*T*_v_), and exposure time during VIPS on the structures and mechanical characteristics of semi-crystalline PVDF. *T*_d_ and *T*_v_ of PVDF have tremendous influence on its structures, as higher values of them enhance phase separation [53]. During polymer composite fabrication, the slower non-solvent intake rate would lead VIPS to prefer polymer crystallization (S–L demixing) over L–L demixing because the former would result in a longer stay of the cast film in the crystallization region without being affected by the latter [54]. A bi-continuous structure of PVDF membrane was created when the dissolving temperature was 32 °C, the modulus was 1.2 MPa, and the crystallinity 60%, according to Li et al. [11]. Peng et al. [12] also observed gradual deformation of the surface of the membrane from a dense to a bi-continuous structure when the vapor temperature increased from 27 to 75 °C at 100% vapor humidity. Good knowledge about the solubility of the polymers and solvent parameters is required to select the vapor required for the phase separation process. Though the process is comparatively faster, it causes fast vaporization of solvents that can leave irregular pore structures. However, this can be tuned by controlling the vaporization speed.

### Non-solvent-induced phase separation process (NIPS)

NIPS involves casting a homogeneous polymer solution on the substrate and then immersing it in a coagulation bath. The diffusion kinetics and mass transfer rate determine the morphology of the phase-separated PVDF [13]. During NIPS, depending on the kind of polymer and the precipitation circumstances used, diffusional exchange between solvent and non-solvent causes the solution to enter a thermodynamically unstable state, resulting in phase separation, either via liquid–liquid (L–L) or solid–liquid (S–L) demixing [55]. For example, Cui et al. investigated the phase diagram of PVDF/[BMIM]BF_4_ at various PVDF concentrations. During 10–50 wt% polymer concentration, L–L phase separation predominates when the temperature of the casting solution declines, and S–L phase separation predominates when the temperature of the casting solution is lower than the crystallization temperature. When the polymer concentration is 20%, the arrow in Fig. 2c depicts the cooling course of the casting solution. A homogeneous casting solution enters the restricted L–L phase separation region when it cools to the cloud point at a high temperature. When the temperature falls below the crystallization temperature [15], the system enters the S–L phase separation region. However, in NIPS, desired morphology and good performance for the polymers require complicated control of the solvent exchange rate by simultaneously varying the dope composition, additives, coagulation medium, quenching bath temperature, and evaporation time [15]. Moreover, the thickness of the polymer film can negatively influence the efficiency of phase separation. In fact, the non-solvent thermally induced phase separation (N-TIPS) method has also been developed to address the drawbacks of TIPS and NIPS methods.

### Polymerization-induced phase separation process (PIPS)

Phase separation in a multicomponent mixture caused by the polymerization of one or more components is known as polymerization-induced phase separation (PIPS) [54, 56]. One or more components become mutually immiscible when the molecular weight of the reactive component increases, resulting in spontaneous phase separation. Thermally induced polymerization or photopolymerization can both cause PIPS [57–59]. The process is triggered by SD, which frequently leads to the creation of co-continuous phases [60]. SD is a phase separation process in which heat transfer instability causes homogeneous blends to separate into more than one phase. The procedure is extensively utilized in thermoelectric, solid-state lighting, polymer electrolytes, composites, membrane construction, and surface pattern forms to regulate the morphology of polymer blends [61]. PIPS is also employed in the preparation of polymer dispersed liquid crystals (PDLCs). This involves the combination of monomers and liquid crystals (LC) to induce polymerization, followed by the two stage splitting of phases. In this case, the matrix phase contains the majority of the polymer, and the other LC phase contains the majority of the LC and the unreacted monomer. In fact, the LC phase appears as discrete droplets in the matrix phase. The cross-links in the matrix, or the vitrification of the matrix in the case of linear polymerization, prevent these droplets from coalescing [62]. In PIPS, various polymer morphologies (Fig. 2d) can be produced by changing the concentrations of modifier, molecular weights of polymers, and curing conditions [63]. However, the process is longer and involves complex polymerization steps.

### Bi-solvent-induced phase separation process

BIPS involves solvents of different polarities, and the polymer is dissolvable in one of them, as in NIPS. For example, Mishra et al. described the bi-solvent phase separation of the *δ*-phase in PVDF by forming the interface between the solvent (acetone) and a non-solvent (water: methanol) [23]^.^ As shown in Fig. 2e, the nanoparticles of *δ*-phase separate from the solvent at the interface of the good solvent and the non-solvent (represented by a rectangular section in Fig. 2e). The *δ*-phase nanoparticles separate from the solvent as a precipitate at the bottom of the tube. BIPS is relatively fast and simple process and can be adopted for different polymers. But for the PVDF-based polymers this method is less widely used because of the specific requirements of phases, related to piezoelectric properties. Since the *β*-phase is the prominent phase contributing to the piezoelectric properties in PVDF polymers, strategies to separate this phase are more demanded.

In general, phase separation is also influenced by the presence of nanoadditives within the polymers. In composites, nanomaterials cause structural reinforcing, by interconnection throughout. This restricts the free polymer chain movement during phase separation. Therefore, nanocomposites must be thoroughly studied for their nanomaterial properties, particle–solvent interaction, particle polarity, and particle–polymer interactions before deciding the type of phase separation.

## Phase separation in piezoelectric nanogenerators

**Fig. 3:**
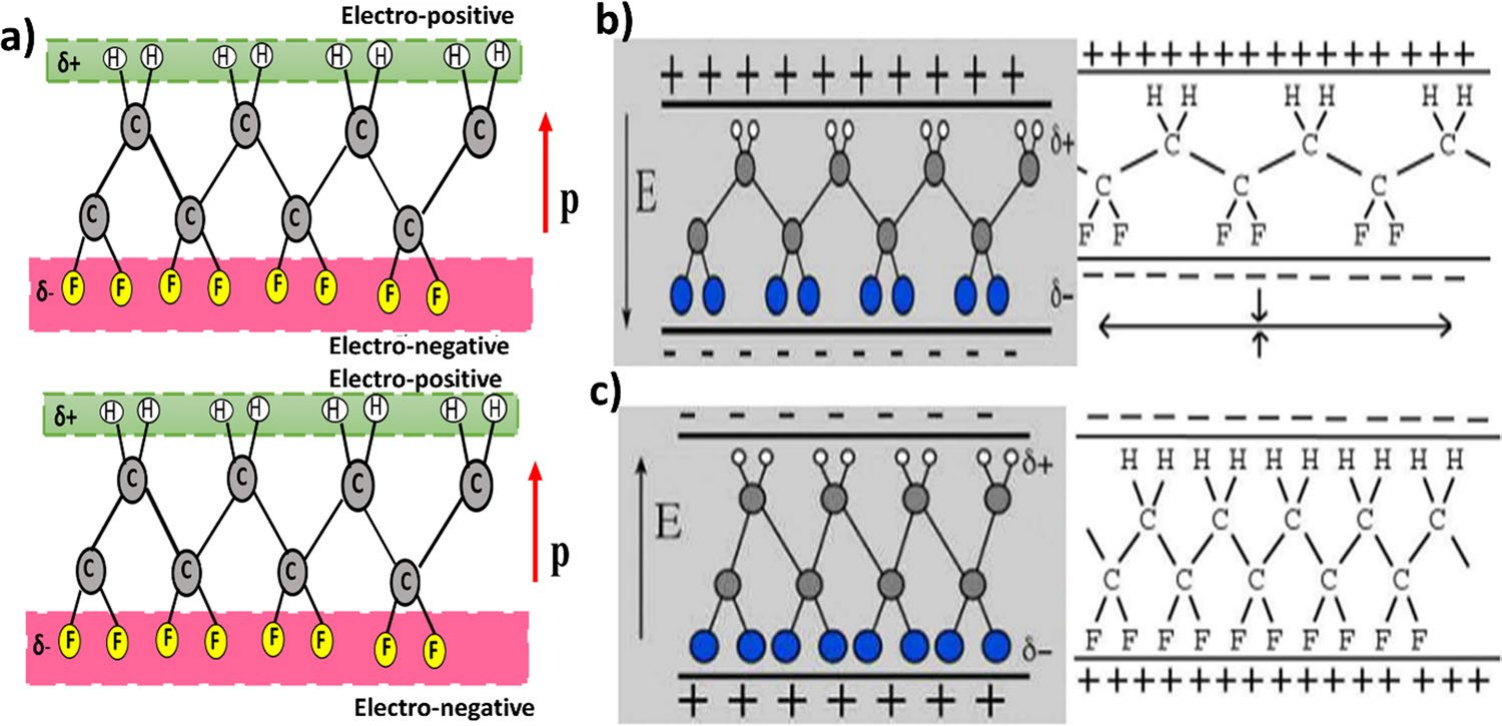
Schematic representation of piezoelectric effect in PVDF due to *β*-phase. **a** Dipoles with charge separation created within *β*-phase. **b** Stretched sheet, with the electric field facing in the opposite direction as the poled direction. **c** Reduced sheet length, and the electric field is equal to the poled direction [64]. Reprinted under creative common license

In the crystalline structure of CH_2_CF_2_ chains of PVDF, *β*-phase is made of huge, translucent sheets that are stretched and poled (Fig. 3). When the sheet is held up to the light, the stretch direction is the direction in which most of the carbon chains run and is visible to the human eye. The poled direction “p” points to the sheet’s top or bottom. The net positively charged hydrogen atoms and the net negatively charged fluorine atoms end up on opposing sides of the sheet. This results in a pole direction, as indicated by the arrow in Fig. 3b [64]. When the electricity is applied across the sheets, either they expand along the stretch direction or contract in thickness, depending on the field direction. The effects of interaction between the positively charged hydrogen atoms on the negative side of the electric field and negatively charged fluorine atoms with the positive side of electric field determine the contraction in PVDF sheets (Fig. 3c).

The majority of the research is currently focused on improving the *β*-phase content by the addition of different inorganic ceramic materials, such as graphene oxide (GO) and ZnO, and thus enhancing the piezoelectric output voltage. Out of the various fabrication methods of nanocomposites, electrospinning technology is versatile for *β*-phase formation and advanced applications [64]. Though electrospinning immediately aligns the dipoles in PVDF, facilitating good output voltage, it is a high-cost process due to the required high voltage and is time-consuming. Recently, phase-separated (NIPS) PVDF/graphene composite was coated on commercially available fabric by Xie and co-workers for motion sensing and speech monitoring purposes [34]. With 0.5 wt% graphene, the electroactive phases (*β*/*γ*) became 87%, increasing the material’s piezoelectric characteristics. As the input intensity was raised from 0.05 to 0.45 N, the voltage signal grew linearly from 3 to 18 V with a sensitivity of 34 V/N. With a force of 2 N, the PENG produced a high voltage of nearly 60 V, while the detecting threshold was discovered to be 0.6 mN [34]. Similarly, BaTiO_3_@PVDF-TrFE(TrFE = trifluoroethylene) nanocomposites were also fabricated using NIPS to enhance *β*-phase within the PVDF. The resulting nanocomposites possess effective orientation and an excellent output voltage of 59.5 V, with an output current of 6.5 uA at 100 N [37]. The piezoelectric responses for BaTiO_3_@PVDF-TrFE nanocomposite were higher than the BaTiO_3_ nanoparticles, and PVDF-TRFE matrix. In addition, with NIPS, pure PVDF-TrFE and BaTiO_3_ nanocomposite films produced 2 times and 4.8 times higher voltages, respectively. The authors exploited the flexible nature of BaTiO_3_@PVDF-TrFE to design PENG both in 2D thin film and 3D spring shapes [37].

**Fig. 4:**
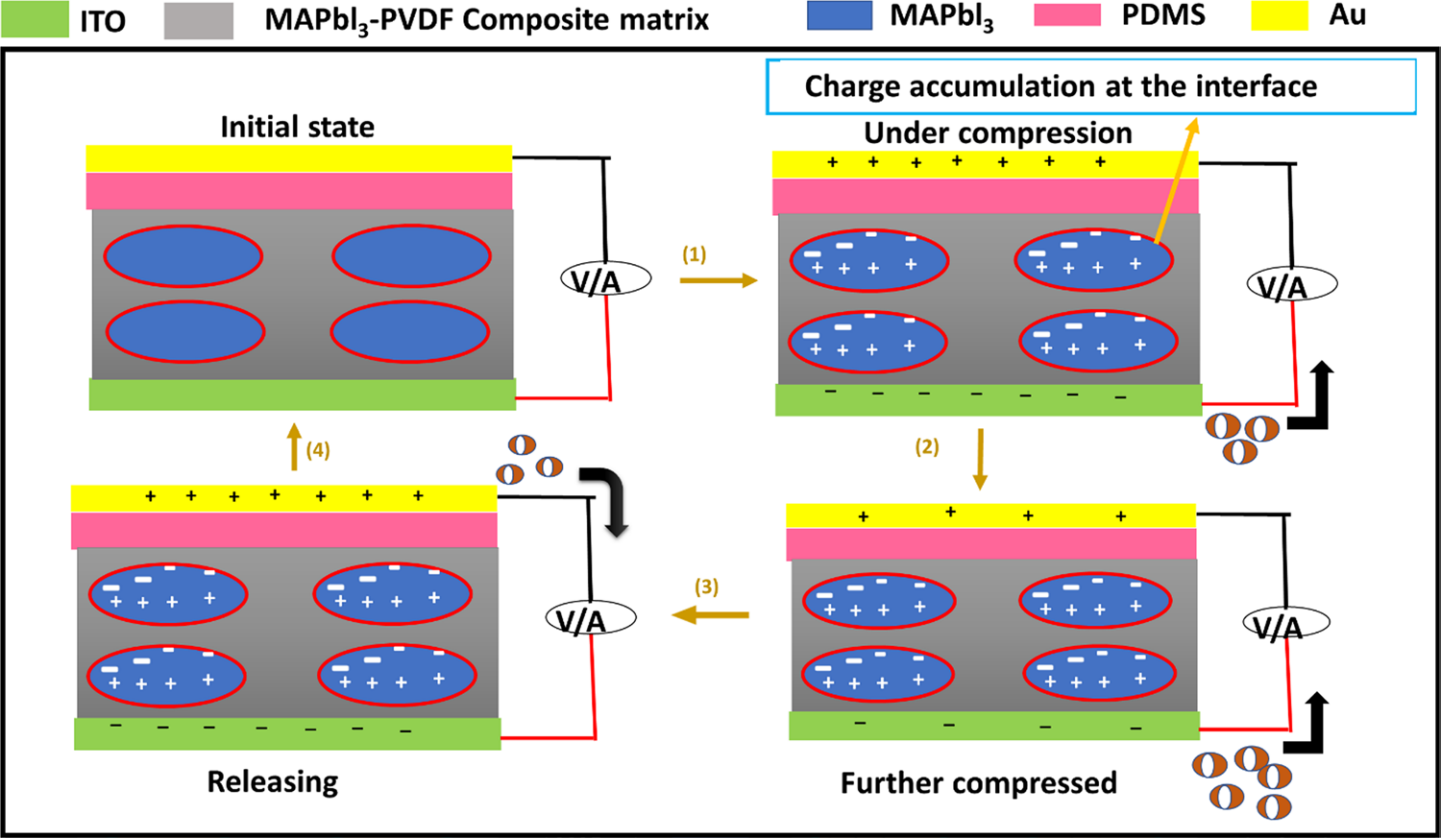
Mechanism of power generation in MAPbI_3_-PVDF nanocomposite-based PENGs [65]. Copyright 2018. Reprinted with permission from Elsevier

Jella et al. fabricated MAPbI_3_/PVDF composite films with spin coating; the mechanism of power generation of the nanocomposite is illustrated in Fig. 4. In the early stages, there are no charges on the electrode surface, and a force applied creates the piezoelectric potential in the crystalline region. MAPbI_3_ generates macroscopic dipoles, accumulating charges of the opposite polarity at the MAPbI_3_/PVDF composite’s interface and surface [65]. However, an agglomeration is observed in the morphology analysis, and larger gaps were formed, especially on 40–50 vol% MAPbI_3_, proving this. This is explained based on the phase separation happening within. The spin-coated samples are pre-annealed, and during this time, a liquid–solid phase separation happens due to the rapid crystallization of MAPbI_3_.

**Fig. 5:**
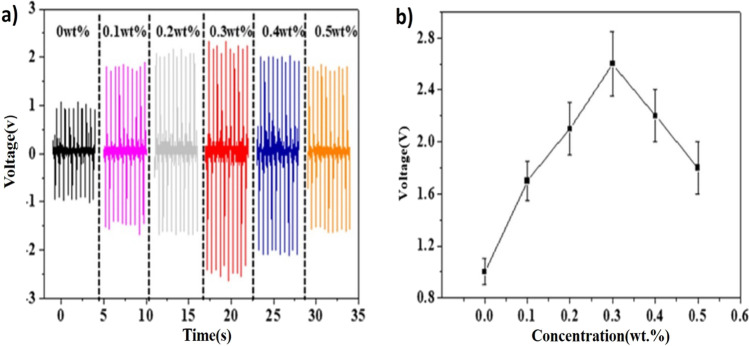
**a**, **b** With varying BNNT concentrations, the output voltage of a P(VDF-TrFE)/BNNTs nanocomposite film-based nanogenerator [66]. Copyright 2019. Reprinted with permission from Elsevier

Ye and co-workers fabricated the PENG, comprising of P(VDF-TrFE) and boron nitride nanotubes (BNNTs). The micro-structured P(VDF-TrFE)/0.3 wt% BNNT, when exposed to 0.4 MPa pressure, exhibited an output voltage of 22 V and a sensitivity of 55 V/MPa, which are 11-fold higher than those of pristine P(VDF-TrFE) film. Synergistic effects from strong piezoelectric BNNTs and a strain confinement effect of the nanocomposite microstructure are credited for the substantial improvement in performance [66]. Several PENGs based on nanocomposite films with varied mass fractions of BNNTs were prepared to examine the effect of BNNT concentration on PENG output performances. The output voltage of PENGs is shown in Fig. 5a, b for varied BNNT concentrations ranging from 0 to 0.5 wt% percent. As the number of BNNTs grows from 0 to 0.3 wt%, the output voltages climb from 1.0 to 2.6 V. Following that, when the concentration increases from 0.3 to 0.5 wt%, the output voltages steadily fall from 2.6 to 1.7 V. The authors claim that P(VDF-TrFE)/BNNTs composites can be effectively used in human body protection from cosmic radiation and also as biomechanical energy harvesting devices.

Very recently, Ban et al. [67] fabricated a CsPbI_3_@PVDF composite by making use of temperature-assisted curing for the spin-coated composite. The particular fabrication strategy separates the filler phase and forms the long-term stabilized black *γ*-phase of CsPbI_3._ This phase of the CsPbI_3_-based PVDF composite performed two times better than the yellow phase of the CsPbI_3_-based composite. A layer-by-layer stacking process was used to adjust the thickness of the composite film. The output voltage of a five-layer black-*γ*-phase CsPbI_3_@PVDF composite PENG was 26 V, with a current density of μA/cm^2^. The output power can be up to 25 watts. In addition, the PENG can be used to charge capacitors via a bridge rectifier and has demonstrated long life in tests with over 14,000 cycles. These results show that inorganic perovskites can be used to build and develop high-performance PENG [67]. However, the phase formation in PVDF and the interfacial interactions between CsPbI_3_ and PVDF are not mentioned in this research. It can be further extended to deeply investigate the phase behavior of PVDF.

**Fig. 6:**
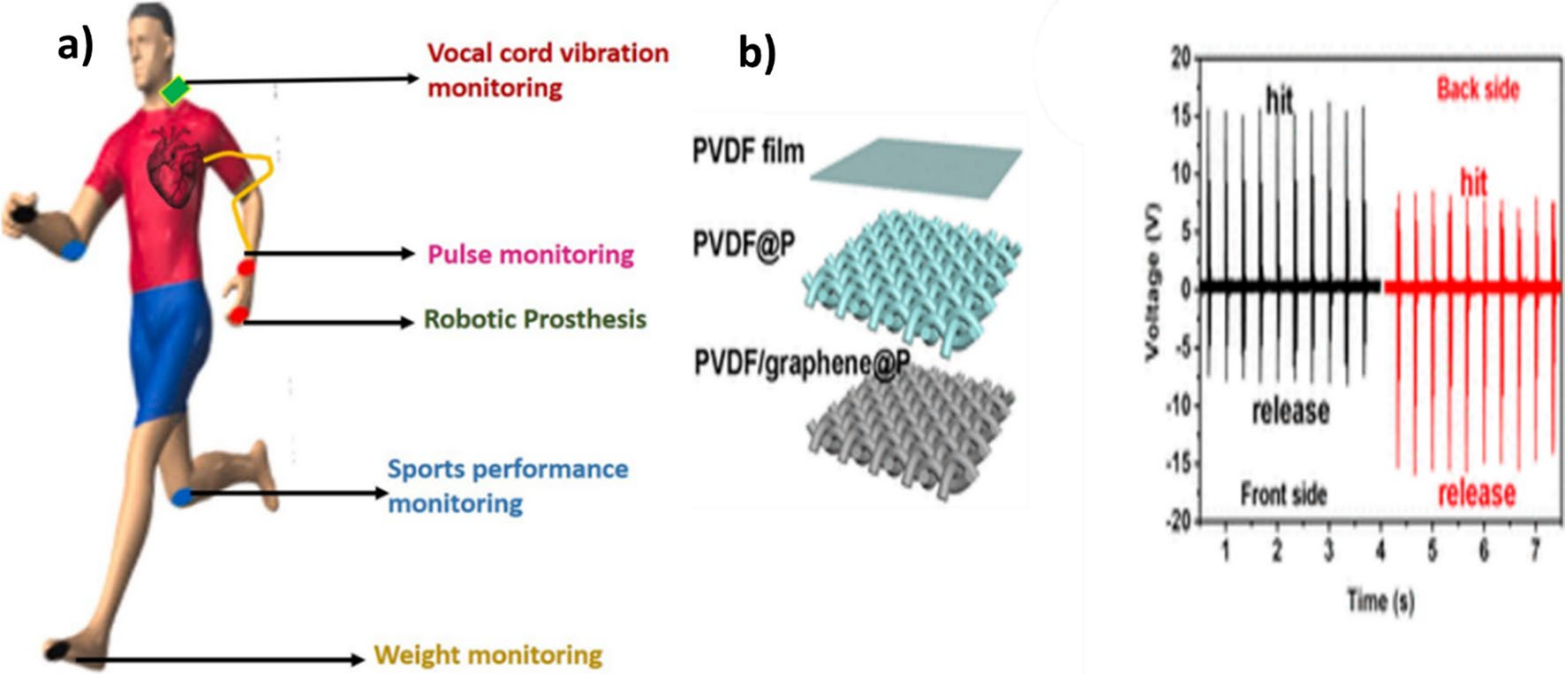
**a** Piezoelectric sensor for health monitoring. **b** PVDF/graphene/polystyrene piezoelectric sensor [34]. Copyright 2018. Reprinted with permission from Elsevier

Modern society is hugely dependent on portable electronic devices that can convert mechanical signals into electrical signals in practice for real-time biomedical monitoring and human motion sensing. Piezoelectric sensors [68, 69] of PVDF respond more accurately to external forces than typical resistance-based test system sensors, in a self-powering mode of operation. Mandal and co-workers reported $\delta$-PVDF nanoparticle fabrication using the BIPS method and their outstanding open-circuit voltage capacity and power density. These $\delta$-PVDF nanoparticles were used as biosensors for the real-time monitoring of arterial pulse signals without the necessity of an external power source [70]. Phase-separated PVDF/graphene composite coated on fabric found specific usage in wearable piezoelectric sensors. The sensor possessed a sensitivity of 34 VN^−1^, which is superior to other sensors reported so far. Moreover, it had a low detecting threshold (0.6 mN) that could be exploited to distinguish voices and detect human body motion [34]. As shown in Fig. 6, when the stress is applied to the front side of PVDF/graphene/polyester, the sensor generates corresponding voltage signals. When the piezoelectric sensor is overturned and hits its rear side, the voltages are also reversed. Due to the alignment of PVDF’s –CH_2_–/–CF_2_– dipoles, the greatest voltage on the rear side was practically identical to that on the front side, demonstrating indirect dipole reversibility [71].

Biocompatibility and healing of bone-related diseases by PVDF PENGs were illustrated by Gong and co-workers [72]. They fabricated a nanocomposite scaffold by doping Ca–P–Si into PVDF by the phase separation hydration (PSH) method. They identified the PSH method as producing a better piezoelectric scaffold than that of raw bone (3 pC/N vs. 0.7 pC/N). In addition, the scaffold was structurally compatible (7 MPa) with cancellous bone and had sufficient porosity (45%) to allow osteoblast infiltration and bone growth. The constructed CPS-PVDF scaffold was biocompatible with osteoblast cells and stimulated osteoblast re-differentiation, as revealed by in vitro biocompatibility tests.

**Fig. 7:**
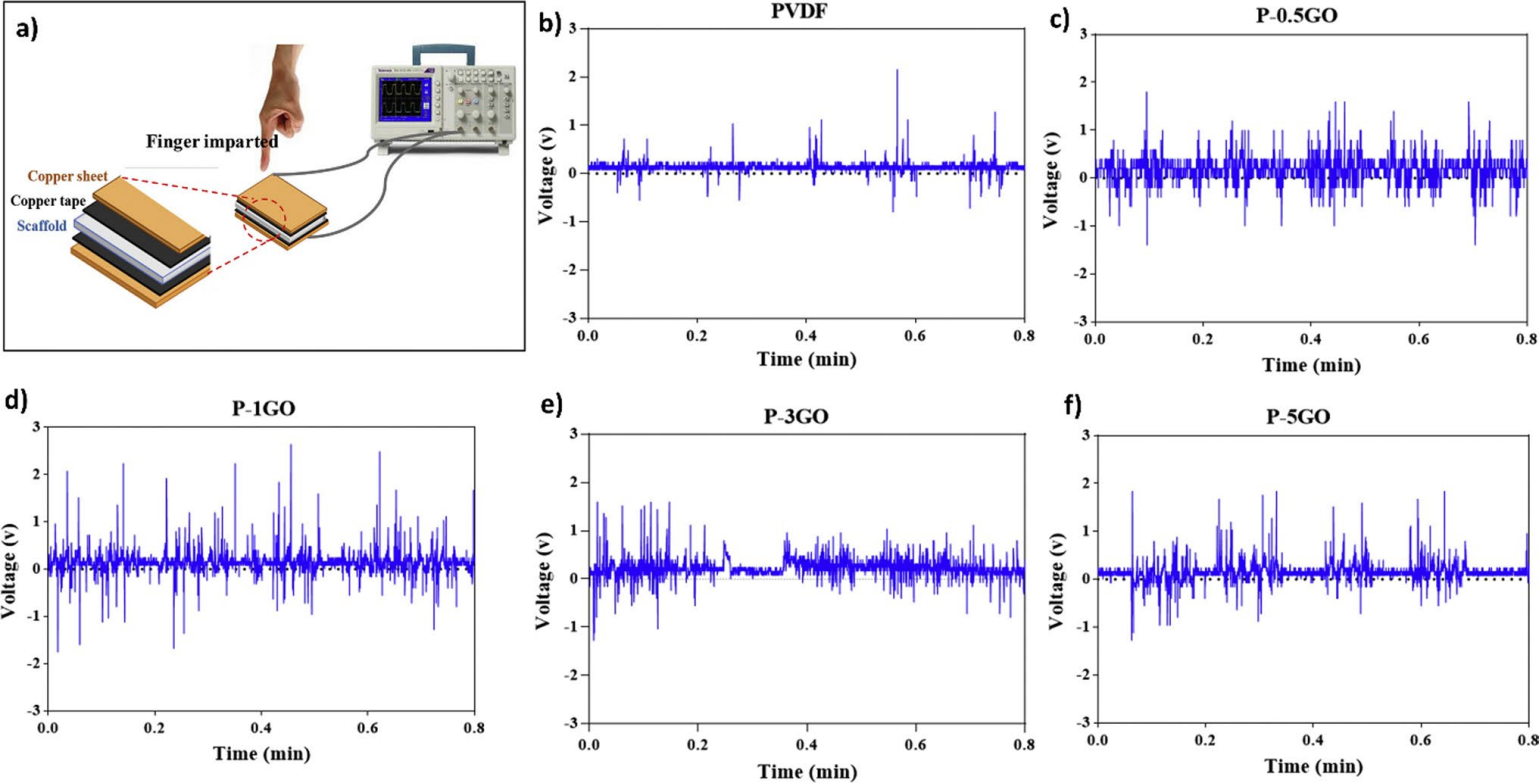
**a** Representation of piezoelectricity measurement by PVDF-GO scaffolds with various GO contents. **b** 0 wt% (PVDF), **c** 0.5 wt% (P-0.5GO), **d** 1 wt% (P-1GO), **e** 3 wt% (P-3GO), and **f** 5 wt% (P-5GO) (P-5GO) [73]. Copyright 2019. Reprinted with permission from Elsevier

GO nanosheets are introduced to PVDF by NIPS to increase the hydrophilicity, water absorption, water flux, and mechanical properties of the scaffolds. Increasing the concentration of GO to 3 wt% significantly increased the tensile modulus and strength of the PVDF scaffold from 8.1 1.4 and 0.8 0.2 MPa to 17.0 3.7 and 1.4 0.4 MPa, respectively. GO nanosheets also improved the phase fraction, piezoelectricity, and electrical conductivity of all nanocomposite scaffolds. The phase-separated composite promoted cell proliferation more effectively than PVDF scaffolds, depending on the GO content. The phase-separated PVDF-GO scaffold with its four internal longitudinally aligned channels easily transforms into a nerve guidance tube [73]. Based on Karan’s work [74], electrode–composite–electrode stacks using carbon tape as an electrode were also developed [73]. As shown in Fig. 7, piezo-responses for the PVDF-GO scaffolds were recorded by repeatedly applying mechanical energy to the top sample surface with finger tapping (Fig. 7a). Upon tapping, electric charge distributes on PVDF-GO scaffolds, resulting in an electrical potential shift between the two electrodes and an open-circuit voltage with positive and negative amplitudes. GO concentration (0.5 wt%) increased the open-circuit voltage (1.4-fold) and decreased the electrical conductivity (1.7-fold), since it influences *β*-phase formation. However, higher concentrations negatively influence the piezoelectric properties. In fact, the NIPS enhances the adhesion, spreading, and proliferation of composite cells and further improves the *β*-phase [73]. Comprehensive data for the phase-separated PENGs are provided in Table 1.

**Table 1:** Phase-separated composites for piezoelectric applications

Polymer	Filler	Phase separation	Output voltage	Output current density	Power density	Advantages	References
PMMA/PVDF-TrFE	BaTiO_3_	*β*-phase/electrospinning	12.6 V	1.30 A	4025 W	High compatibility, Young’s modulus, and strong stress transmission at the interface	[59]
PET/PVDF	Graphene	VIPS	300 V	72 A	4016 mWm^−2^	High stability and durability	[38]
PVDF/TrFEE	BaTiO_3_	NIPS	59.5 V	6.5 µA	4.25 W	Flexible as 2D thin film and 3D spring	[37]
PVDF	KNN	*β*-phase/electrospinning	1.9 V	–	–	Lead-free PENG, unique energy harvesting	[75]
PVDF	Alo-rGO	VIPS	36 V	0.8 µA	36 mWm^−2^	High stability and durability	[71]
PVDF	CsPbI_3_	*γ*-phase	26 V	–	25 W	Capacitor charging via bridge rectifier and durable over 14,000 cycles	[67]
PVDF	Ca-P-Si	*β* and *γ* phases	–	–	–	Biocompatible and used in healing bone diseases	[72]
rGO-PEI/PVDF-HFP	rGO	*β* phases	2 V	100 nA		Bio-friendly drug delivery devices	[76]
PVDF/PANI	rGO	*β* crystalline phases	10.60 V	–	–	High voltage	[77]
PVDF	Graphene	*α*To F(*β*) phase separation	7.9 V	4.45 µm	–	High power, light LEDs for 30 s	[78]
PVDF	CdS/rGO		4 V	–	–		[79]
PVDF	ZnO	*α*–*β* phase separation	0.354 V			Enhanced electron transfer to the film top	[80]
PVDF	rGO-Ag	*β* and *γ* phases	18 V	1.05 µA	28 Wm^−3^	Flexible, lightweight, low cost, no external cooling required	[81]

**Fig. 8:**
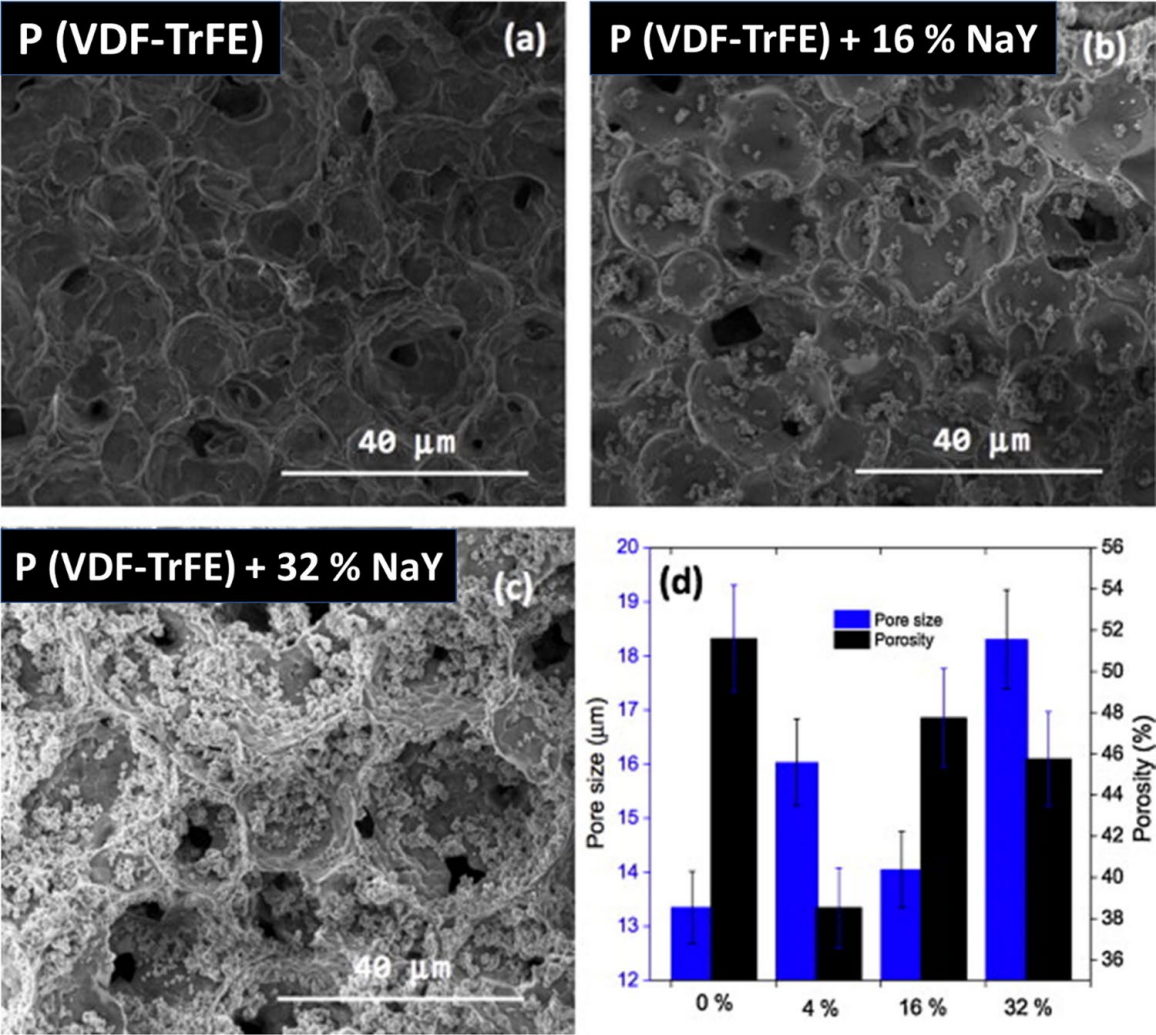
SEM images of PVDF-TrFE and its composite with different zeolite contents (**a**–**c**). Porosity and pore size as a function of filler content (**d**) [83]. Copyright 2015. Reprinted with permission from Elsevier

Macroporous P(VDF-TrFE)/NaY zeolite membranes with a filler content up to 32 wt% were introduced as a suitable platform to release ibuprofen [82]. The porous microstructure of the composite as illustrated in Fig. 8 is described by a liquid/liquid spinodal breakdown followed by polymer crystallization, and it has a significant impact on the membranes’ absorption capability. The ibuprofen was encapsulated inside the pores using a physical deposition process, and the NaY zeolite was immobilized on the surface of the pores of polymer P(VDF-TrFE) during the polymer crystallization process and solvent extraction. The polymer composite membranes with zeolite content of 16 and 32 wt% were used to monitor the release of ibuprofen, and both membranes exhibit good mechanical performance, with a large initial release followed by stabilization, and the membrane with a 32% NaY release has more than double the ibuprofen level of the one with 16%. The formation of such interconnected pores during liquid–liquid phase separation was previously reported for PVDF-TrFE by Ferreira et al. [83]. They explained it using the PVDF-TrFE/DMF phase diagram, which demonstrated the initial volume fraction at different temperatures. In spinodal liquid–liquid phase separation conditions (volume fraction and temperature), a homogeneous and well-organized porous structure forms with spherical interconnected pores and pore walls with adhered spherical polymer crystals. Such porous architecture has numerous applications, including filtration, drug delivery, scaffolds, etc., other than the piezoelectric nanogenerators.

## Phase separation in triboelectric nanogenerators

The triboelectric effect is defined as contact-induced electrification through friction when a material becomes electrically charged after it is contacted with another different material [84]. Upon contact between two different materials, charges, electrons, ions or molecules, jump from one material to the other to equalize their electrochemical potential. When these materials separate, some of the bonded atoms tend to keep extra electrons; however, there is a tendency for some to give them away, which may produce a triboelectric charge on surfaces [85]. The ability to gain or lose electrons depends on the material’s polarity and the morphology of the surface. The sign of the charges carried by the materials depends only on their relative polarity in comparison with the material with which they are in contact. The triboelectric effect has been used for a long time for various applications, such as Van de Graaff generators invented in 1929 and the Wimshurst machine in 1880. These two machines use the accumulated static charges. Nowadays, the world is following the miniaturization trend, and TENGs are considered one of the advanced technologies for sustainable power generation and advanced applications like sensors, robotics, and artificial intelligence [86]. However, its performance depends on the surface charges created and different modifications, in addition to the mode of operation, indicating the need to explore more of this powerful technology.

**Fig. 9:**
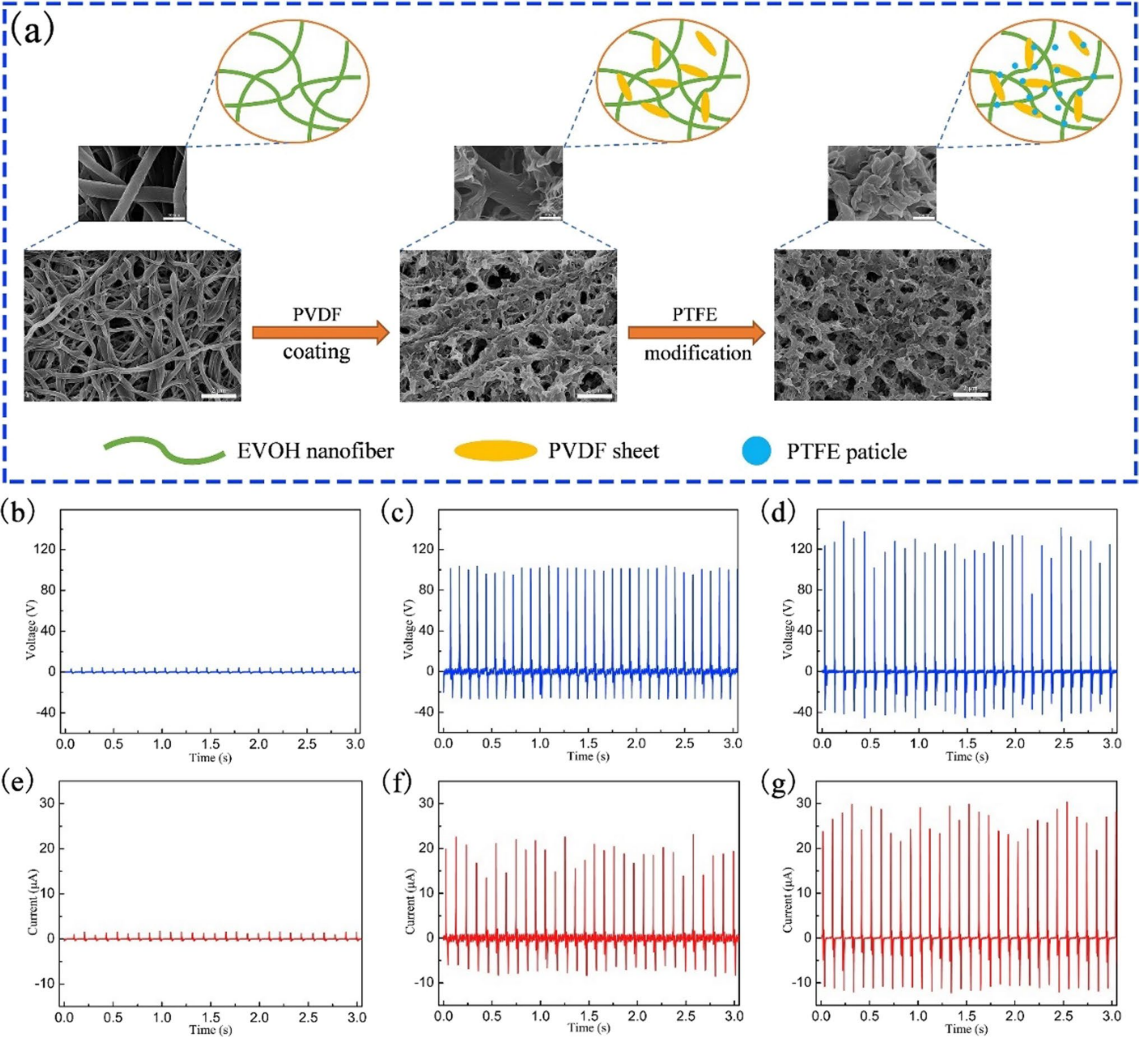
**a** Schematic representation of the post-fabrication modification of PVDF and PTFE nanofiber surfaces and the corresponding FE-SEM images. **b** Voltage output and **e** short-circuit current for the smooth EVOH nanofiber membrane/Al foil triboelectric pair, **c** Voltage output and **f** short-circuit current by the triboelectrically coupled EVOH nanofiber membrane with two layers of PVDF and Al foil, **d** Voltage output and **g** short-circuit current by EVOH nanofiber membrane coated with two layers of PTFE/PVDF composite (PTFE: PVDF = 3:12) and Al foil [4]. Copyright 2019. Reprinted with permission from Elsevier

Phase separation modifies the surface features of polymer nanocomposites and improves triboelectric power generation. NIPS was used as a post-fabrication modification method for the thermoplastic EVOH nanofiber membranes by Yan et al. to maintain the nanostructure without changing its crystal form and to enhance the tribo-electronegativity [4]. When modification is done by the PTFE/PVDF mixture, PTFE has the strongest tendency to gain electrons, opening up the prospect of improving output performance. Figure 9 shows the surface morphologies of the fibers before and after modification, along with their output performances. Smooth, thin, and porous EVOH fibers give 4.3 V (Fig. 9b) and 1.7 μA (Fig. 9e) upon triboelectric test; however, PVDF secondary nanosheet formation on the EVOH improves the voltage. Respective output voltage and current of 104.5 V and 23.6 μA are produced by the PVDF double-layer coating (Fig. 9c, f) facilitated by NIPS. When PTFE is included in the coating, again by phase separation, the output voltage and current become 145.5 V (Fig. 9d) and 30.04 μA (Fig. 9g), respectively. This can be attributed to the porous structure and increased contact area within the PTFE due to the phase separation process.

**Fig. 10:**
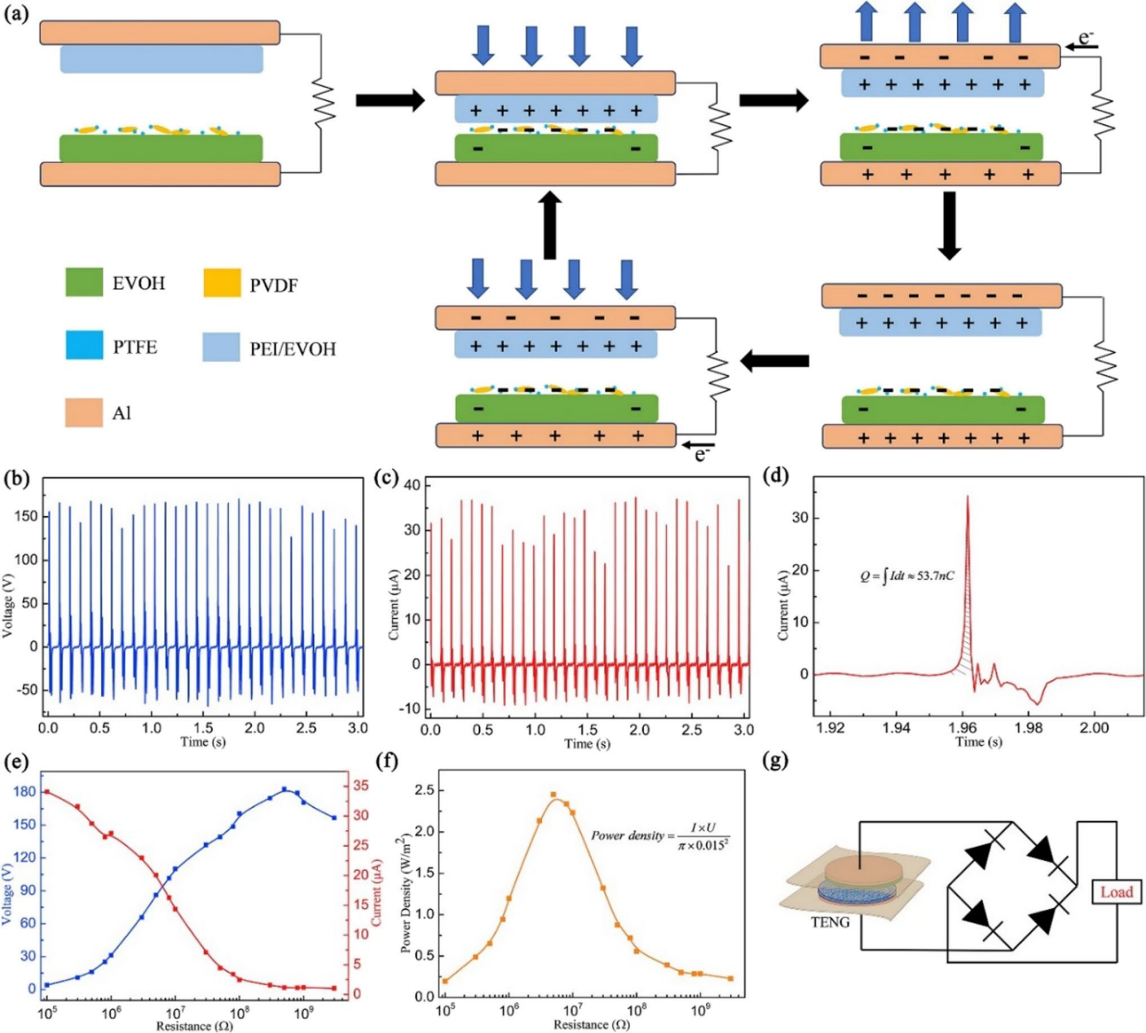
**a** Working principle of TENGs in one compression and release cycle. **b** Open-circuit voltage, **c** short-circuit current, and **d** corresponding transferred charges. Dependence of **e** output voltage and current and **f** instantaneous power density on different load resistances. **g** Prototype power-supply diagram by the TENG with a rectifier circuit [4]. Copyright 2019. Reprinted with permission from Elsevier

When the PTFE/PVDF composite is coupled with the EVOH nanofiber as the triboelectric pair, respective voltage and current of 170.5 V (Fig. 10b) and 37.4 μA (Fig. 10c) are obtained. The mechanism represented in Fig. 10a shows the charge transfer happening in the two layers with a considerable difference in electron affinity. Upon pressing and subsequent release, a potential difference is created between the layers. According to Fig. 10d, the transferred charge generated is 53.7 nC, and further investigation is carried out by connecting the TENG with known resistors. With load, the output voltage increased (Fig. 10e), reaching a maximum instantaneous power density of 2.45 W/m^2^ (Fig. 10f) at 5 MΩ. The researchers could make a sequence of letters (lighted) “MSE” when the external loads were replaced by green LEDs and upon repeated finger tapping on TENGs [4]. The prototype of the power supply diagram is shown in Fig. 10g.

**Fig. 11:**
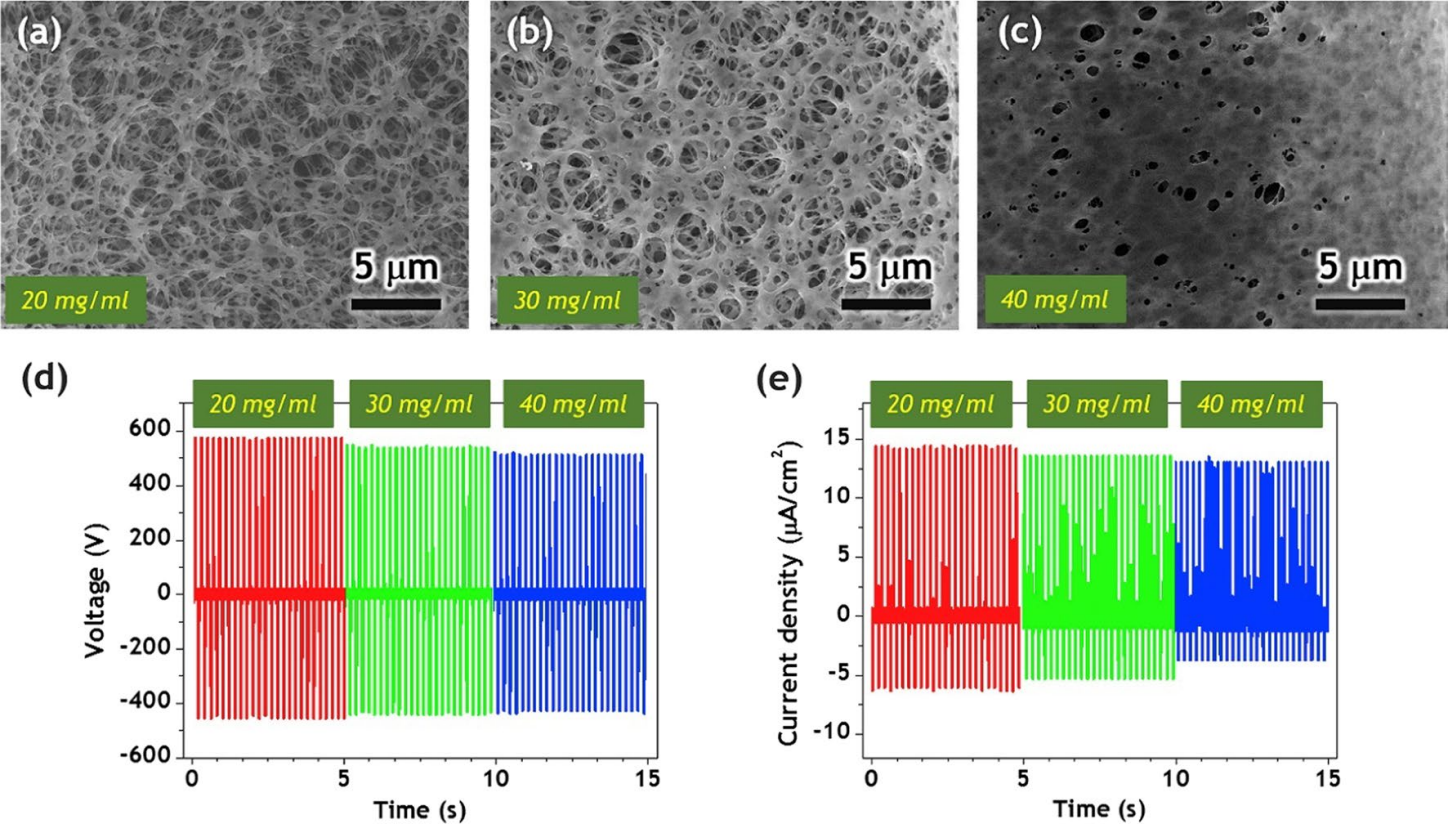
Nanosponge surface morphologies prepared with various polymer concentrations (**a**–**c**). **d**–**e** The effect of NP@Cu-mesh hybrid porosity on the performance of nest-inspired TENGs [87]. Copyright 2020. Reprinted with permission from Elsevier

Bui et al. created a convex-patterned PDMS film for a high-power TENG device using a PMMA mold by evaporation-induced phase separation [87]. Methanol and water were used in this work as the primary and secondary non-solvents in a quaternary solution, respectively. Controlled spinodal breakdown of the quaternary solution is credited as being the mechanism for the formation of the nanosponge PMMA, as shown in Fig. 11. THF has a higher evaporation rate than the combination of methanol and water, which causes an increment in the amount of non-solvent in the solution when the solution is exposed to air. Spinodal decomposition has then been divided into a polymer-rich phase and a non-solvent-rich phase as a result of the gradual enrichment of non-solvent. By aggregating polymers in the polymer-rich phase during phase separation, the polymer matrix can be created, whereas the non-solvent-rich phase develops holes when liquids evaporate. It should be emphasized that the method used to carry out the phase separation, wherein thermodynamic instability and precipitation kinetics are important considerations, determines the resultant porous structure [87]. As the highly porous nano-PMMA sponge generated inside the Cu-mesh holes is lower than the Cu-mesh surface, the strong metal mesh can effectively protect the nanosponge from external impact. The NP@Cu-mesh TENG’s modest drop in output voltage may, however, be due to a partial breakdown of the polymer film because PMMA fragments may have adhered to the PDMS surface, which would have reduced performance through screening [87].

**Fig. 12:**
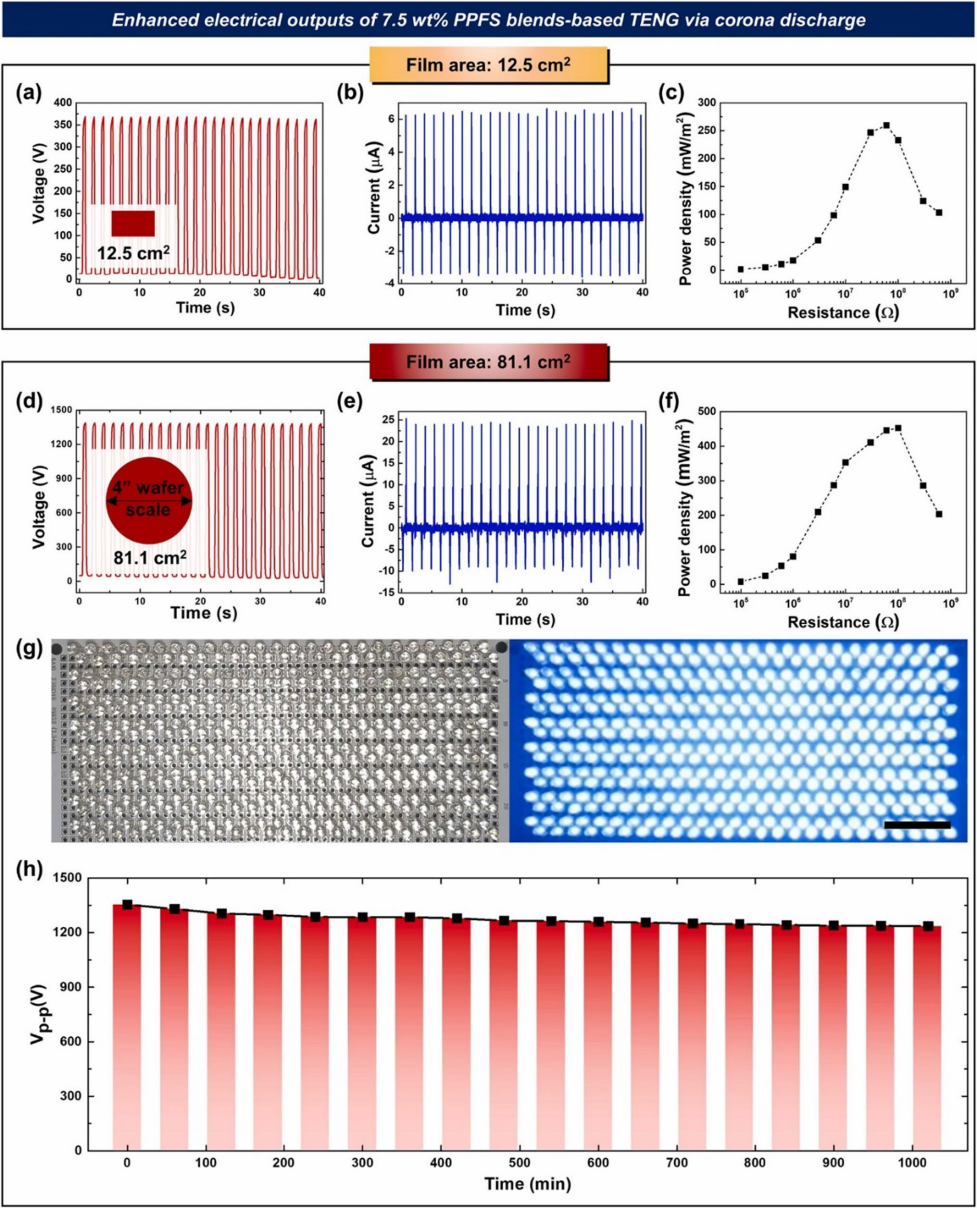
Triboelectric performance of a corona-treated TENG based on a 7.5 wt% PPFS blend. **a** Open-circuit voltage and **b** closed-circuit current **c** Power density as a function of external load resistance; TENG of 12.5 cm^2^ size. **d** Open-circuit voltage and **e** Closed-circuit current and **f** Corona discharge power density **g** Illumination of 400 series-connected blue LEDs (3.3 V/ea), scale bar shows 2 cm and **h** Long-term voltage measurements from a large-scale TENG of 81.1 cm^2^ size after corona discharge on 7.5 wt% PPFS blend [39]. Copyright 2022. Reprinted with permission from Elsevier

Chen et al. combined the dynamic of hierarchical hydrogen bonding with phase separation-like structure to strengthen the fracture energy and toughness of poly(dimethyl siloxane) PDMS for high-performing TENGs. A typical conductive layer of hydrophilic polymer is sandwiched between the PDMS layers [87]. Due to the phase separation-like structure, the composite achieves great strength, toughness, and stretchability, in addition to triboelectric power generation. Furthermore, Choi et al. illustrated the phase separation of fluorine-rich surfaces based on the difference in their surface energy deprived of the toxic fluorine gas. This process helped to synthesize an extremely negative polymer material for its subsequent application in a TENG [88], as demonstrated in Fig. 12. A diminished edge effect is the cause of the large-scale TENG’s better power density. It is well known that as TENG device size increases, the edge impact of CS-mode TENGs decreases. These findings showed that the large-scale, toxic-gas-free synthesis of very negative triboelectric polymers is made possible by the phase separation of fluorine-rich polymers from sulfur polymer matrices at air interfaces [88]. An overview of the phase-separated TENGs reported so far in the literature is tabulated in Table 2.

**Table 2:** Phase-separated composites for triboelectric applications

Polymer	Filler	Phase separation	Triboelectric voltage output (V)	Output current	Power density	References
PVDF	EVOH nanofibers	NIPS	145.5	30.04 μA	2.45 W/m^2^	[4]
PPFS	DIB	TIPS	362.8	6.6 μA	259.7 mW/m^2^	[38]
PLA	GO	NIPS	170	56 μA	3.25 mW/4cm^2^	[39]
PVDF	ZnSnO_3_	Phase inversion	520	2.7 mA	520 V/2.7 mA m^2^	[89]
P-PDMS	D-CNF	Solvent evaporation induced	60.6	7.7 μA	2.33 W/m^2^	[36]

## Phase-separated composites for dual applications

**Fig. 13:**
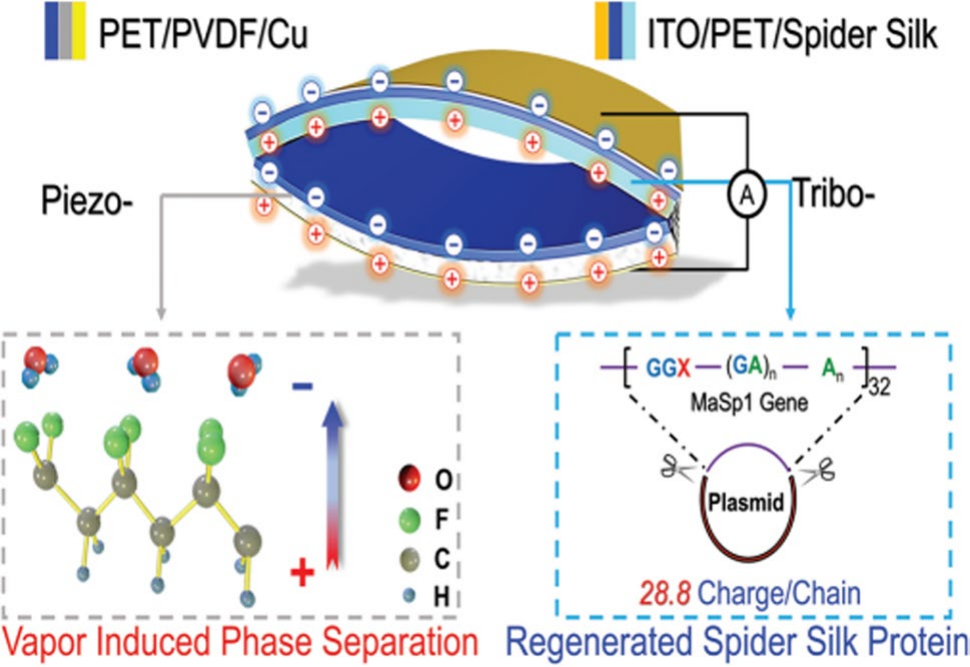
Schematic representation of TPNG model of VIPS PVDF (gray dashed box) and regenerated spider silk protein (blue dashed box) [38]. Copyright 2020. Reprinted with permission from Wiley

A few studies are also reported in which the developed nanogenerator shows dual performance. This means the nanogenerator works both on frequency-dependent mechanical vibration and through contact electrification. Such triboelectric/piezoelectric (TE/PE) nanogenerators, or TPNGs, demonstrate highly efficient collection and conversion of different types of mechanical energies, such as body movement. This can sometimes due to the thin-film structure of its active layers in both TENGs and PENGs, which permits easy integration into preferred configurations for specific applications [38]. Since PENGs require larger mechanical strain and TENGs need a high surface area, innovative structural designs are necessary to design a TPNG that can produce triboelectricity and piezoelectricity at once. Though these nanogenerators are distinguished by a direct energy superposition, they suffer from various disadvantages in functionalization, wearability, and output properties [39]. Huang et al. [38] created a TPNG using an environmentally friendly VIPS from graphene-reinforced PVDF using a self-matching approach. They used spider silk to increase the potential difference with PET/PVDF-graphene and make electron loss easier. Electrons in the specific protein molecular orbits of spider silk transfer to PET empty orbits, proportionally depending on the difference in potential well depths of the two materials. At the same time, the PVDF can change the surface electron potential of contact materials upon mechanical vibration. Figure 13 schematically illustrates the VIPS process for obtaining the nanogenerator. The method is safe, energy-saving, and pollutant-free and is applied to spin-coated PET/PVDF layers. During this process, the PVDF polarizes in the direction from the substrate to humid air (the gray line), and the 0.25 wt% graphene introduced influences the external voltage.

**Fig. 14:**
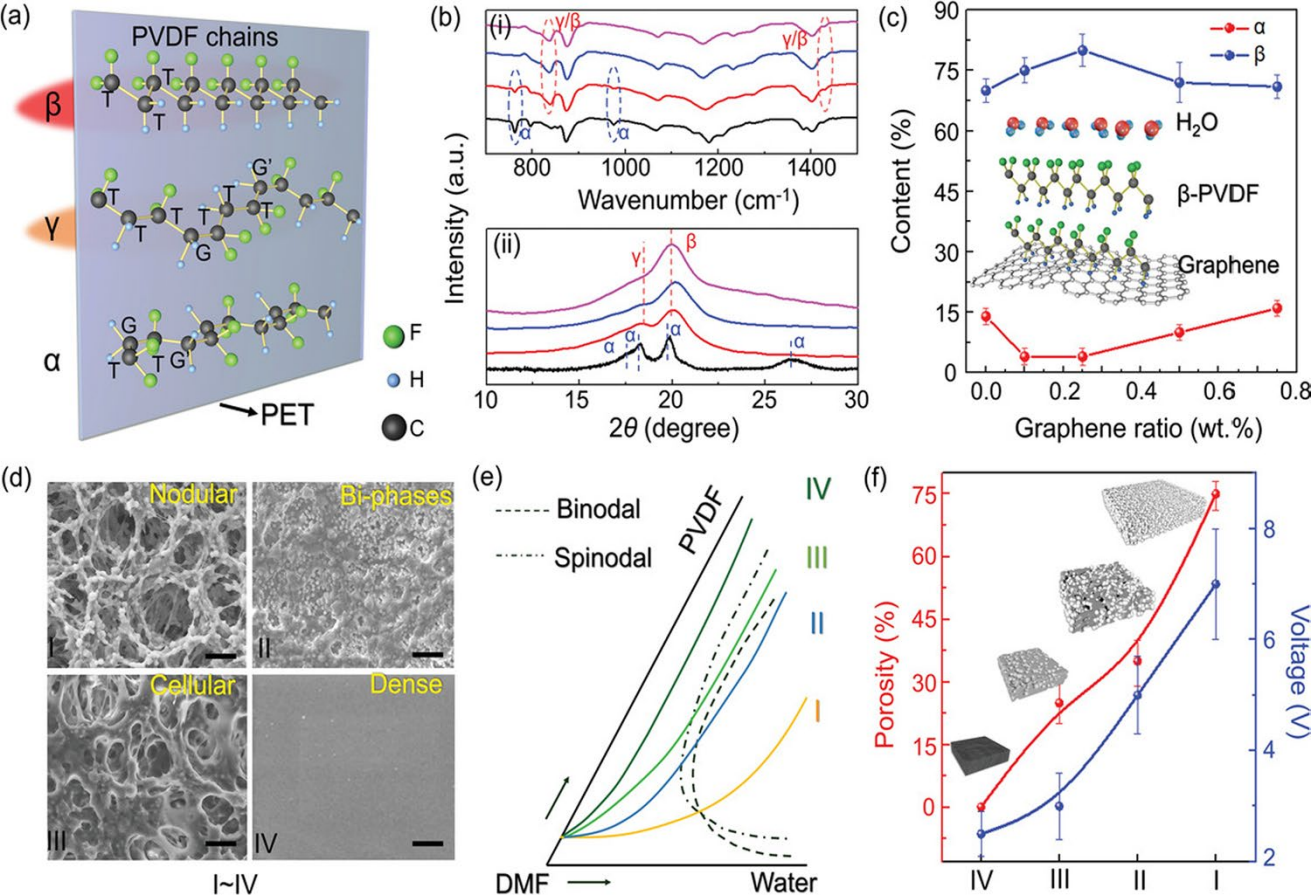
**a** 3D model of various PVDF configurations and their effect on PET surface potential differences. (Color depth corresponds to PET modified intensity.). **b** FTIR spectra (1) and XRD results (2) for raw powder (black curve), natural-evaporated PVDF membranes (red curve), phase-separated PVDF film (blue curve), and graphene doped PVDF film (pink curve). **c** Deconvoluted XRD; phase contents with different graphene doping ratios **d** SEM images of phase-separated PVDF film morphologies (I: nodular; II: bi-phases; III: cellular; and IV: dense structures). **e** Isothermal ternary phase diagram of PVDF/DMF/H_2_O **f** porosity of I-IV structures and the voltage signals associated [38]. Copyright 2020. Reprinted with permission from Wiley

Figure 14 further explains the voltage-generating mechanism of the PE/TE NG. Out of the various crystalline phases of PVDF: trans-gauche–trans-gauche (TGTG′) *α*-phase, all-trans (TTT) planar zigzag *β*-phase and T3GT3G′ *γ*-phase, the latter two are mainly contributing to piezoelectricity, and a proper optimization (Fig. 14a) generates better functioning of PET. The advantage of VIPS in enhancing the *β*/*γ* phase is clear from the FTIR spectra provided in Fig. 14b. The influence of graphene and VIPS on polarizing PVDF is also clear from the XRD provided in the same figure. With 0.25 wt% of graphene, the crystalline *β*-phase became the highest (82%), and the *α*-phase became the least (4–5%) (Fig. 14c). As expected, the VIPS produces porous nodular, bi-phase, and cellular morphologies along with the dense pore-less ball structure (Fig. 14d) corresponding to the ternary phase diagram for PVDF/DMF/water (Fig. 14e). The higher the porosity, the larger the compressibility, deformation at identical pressure, and the mechanical energy transformation (Fig. 14f).

**Fig. 15:**
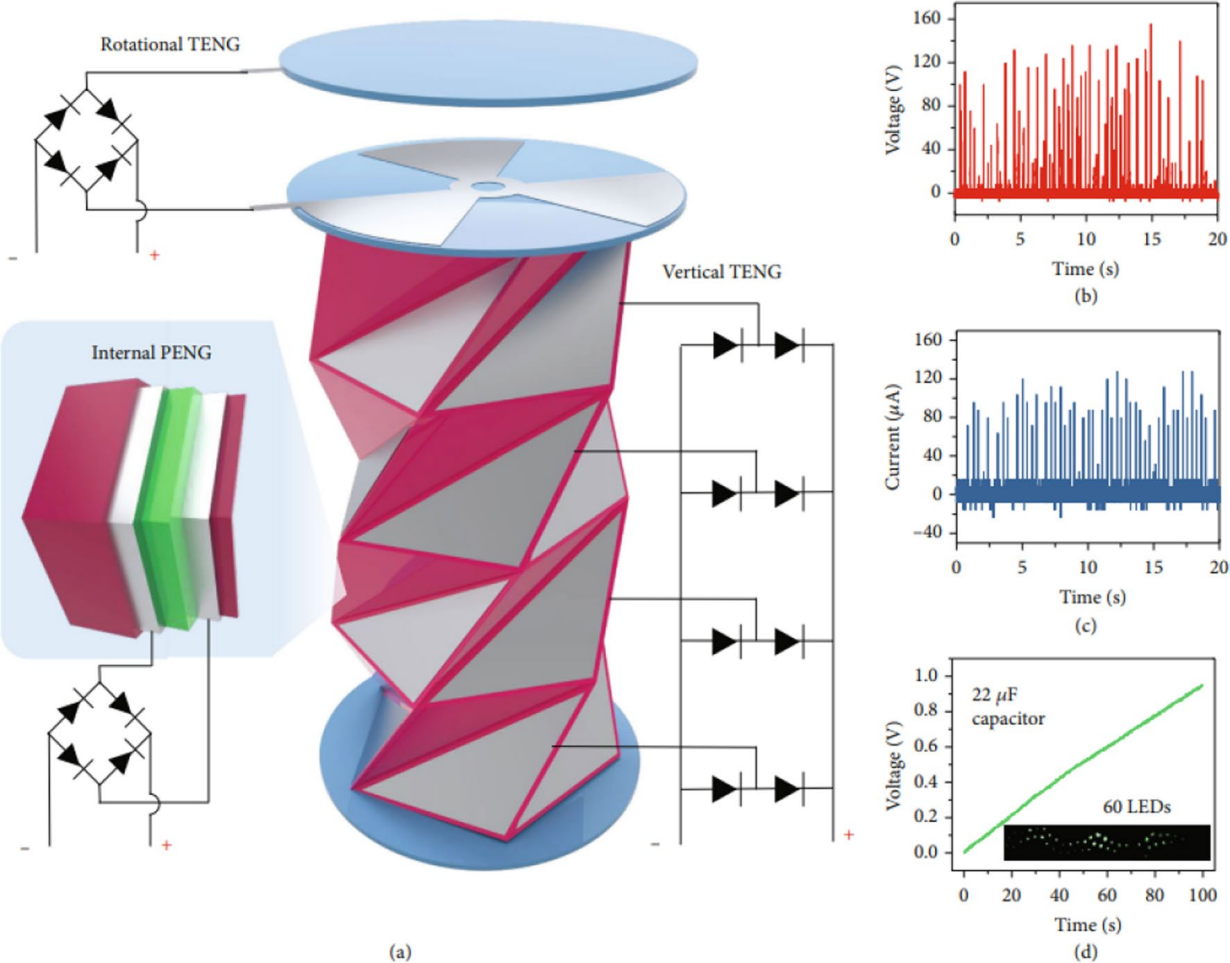
**a** The rectifier circuit connected to the vertical TENG, rotational TENG, and PENG generator. **b** Open-circuit voltage and **c** closed-circuit current output. **d** In a single compression, the nanogenerator charged a 22 F capacitor and powers 60 LEDs [90]. Reprinted with creative common license

Using the NIPS method, Chung et al. [90] created a TPNG with a triangulated cylinder origami-based structure, as represented in Fig. 15. The triangulated cylindrical structure consists of a vertical contact separation TENG on its surface, a rotational TENG on the top substrate, and a PENG on the inner hinge. Figure 15a shows the device connected to a rectifier circuit and the corresponding output voltage (120 V) and current (90 μA). With 6 HZ of vibration, the nanogenerator charged a 22 μF commercial capacitor, powered 60 LEDs, and continuously worked for 48 h. In addition to the power harvesting devices, the TPNGs with high-voltage output and exceptional mechanical flexibility work for a variety of implantable devices, such as monitoring heart, chest, stomach, and bladder signals and detecting stresses brought on by arm, hand, knee, and foot activity [13].

**Fig. 16:**
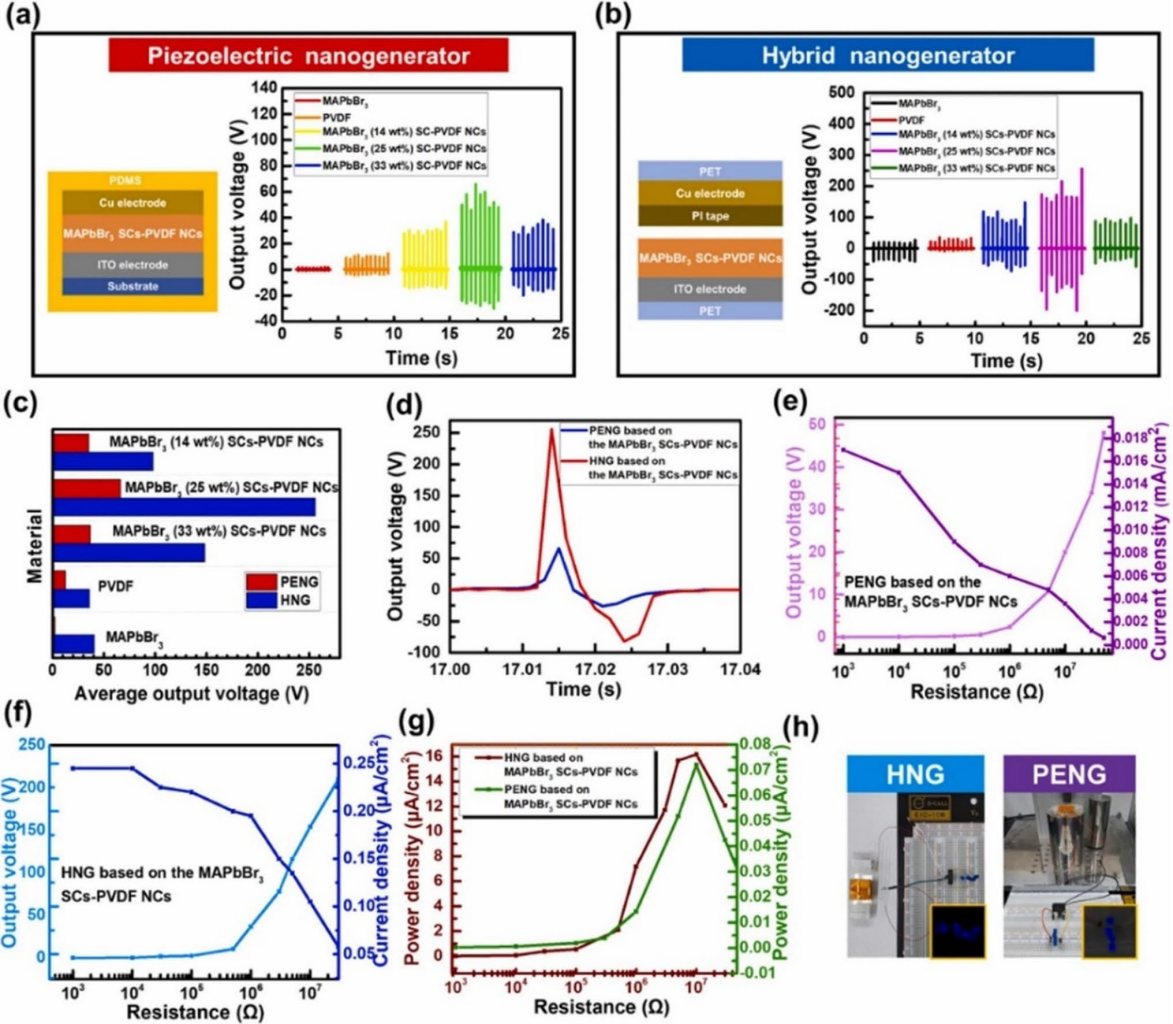
MAPbBr_3_ SCs-PVDF-based **a** PENG and **b** hybrid nanogenerator and the corresponding output voltages with 14, 25, 33 wt% MAPbBr_3_ **c** comparison of voltages **d** Expanded views of the PENG and hybrid generator output voltage waveforms based on 25 wt% MAPbBr_3_ during one cycle. **e** PENG and **f** hybrid generator output voltages and current densities. **g** Power density comparison as a function of external load resistance. **h** Commercial blue LEDs lit with electrical energy generated by a PENG (left) and HNG (right) [91]. Copyright 2022. Reprinted with permission from Elsevier

This review ends with the discussion of a very recent study by Lee et al. [91] for generating porous PVDF structures by VIPS. The mass fraction, vapor temperature, and exposure time are optimized to achieve ultrathin porous morphology with the least nanoparticle (methylammonium lead tribromide single crystals, or MAPbBr_3_ SCs) aggregation. Through charge creation and accumulation, the PVDF NCs film employed in the HNG effectively harvests energy, as shown in Fig. 16. The very high tribo/piezoelectric coupling efficiency caused by the presence of the MAPbBr_3_ SCs in the porous PVDF is clearly demonstrated by the output voltage comparison. When compared, the hybrid NG facilitated 256 V, almost 3.87 times higher than the PENG. In addition, the power density of the hybrid NG (16.17 mW/cm^2^) was 200 times higher at a load of 10 MΩ. However, the MAPbBr_3_ SCs were used in 25 wt%, which was higher for a composite, than expected.

Studies prove the high performance of hybrid nanogenerators by combining the TENG and PENG for powering numerous devices. The biomedical, sensing, and robotics applications [92, 93] of such hybrid nanogenerators need further studies and developments, which are mentioned in the following section.

## Conclusions and future prospects

**Fig. 17:**
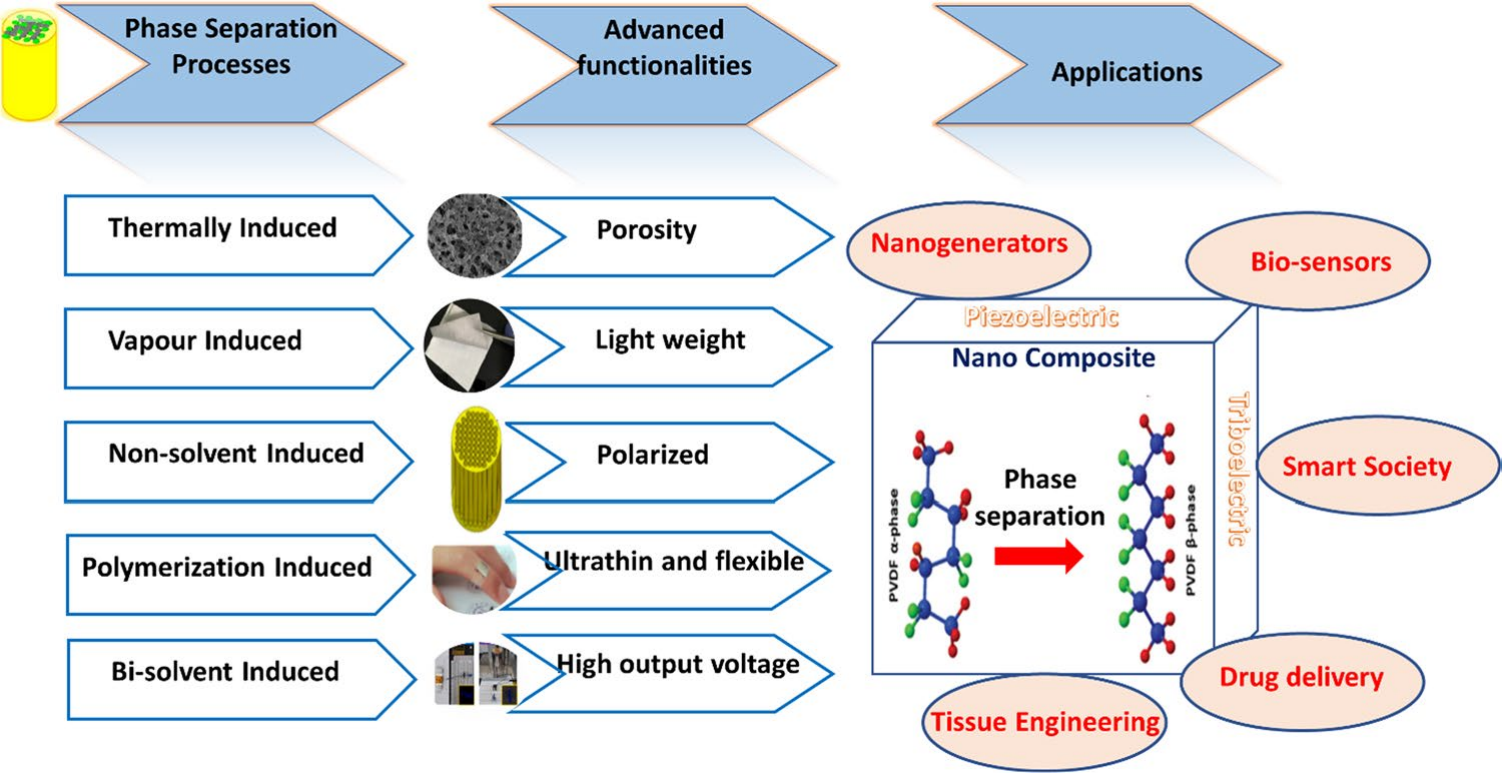
Summary of phase separation and the current/upcoming applications using phase-separated composites

The current study comprehensively reviews the type and mechanism of phase separation processes in polymers and their influence on power generation. Piezoelectric and triboelectric nanogenerators are analyzed for their structural and morphological appearance, polarization behavior, conditions of phase separation, and nature of reinforcement. Phase separation improves the amount of electroactive phases in typical polymers and precisely filters the phase ratios responsible for the piezoelectric mechanism. It also controls the interfacial interactions and maintains high-level porosity for charge distribution, transfer, and electrification. The positive and negative charge centers in the material separate upon a given mechanical stress for a PENG and a frictional sliding force for the TENG to develop a potential difference in the functional layers. This property is further enhanced by the presence of reinforcements or nanoparticles of high electron mobility, good compatibility with the polymer, a wide bandgap, stronger electromechanical coupling, a large aspect ratio, and good dispersibility. Such phase-separated nanocomposites self-polarize and present outstanding output voltages. Developed composites and devices find numerous applications by integrating into wearable and implantable sensors for self-powered gesture detectors, in vivo cardiac monitors, artificial muscles, e-skins, and soft robotics. The superior performance of the PENGs, TENGs, and hybrid nanogenerators provides green and human-friendly approach to scavenging energy from the environment in the current and future Internet of things (IoT) era. The unique structure allows interlayer potential accumulation and rapid stress release for ultrafast response time, excellent sensitivity, durability, and mechanical stability. Piezoelectronic and triboelectronic wearable sensors are used for human motion monitoring, physiological signal monitoring, and personalized health monitoring in bionic robotics and biomedicine. For all these applications, phase separation ensures lightweight, high flexibility, and high-voltage electrical properties. Figure 17 summarizes the advantages of phase separation and the futuristic applications related to this topic.

Despite the significant application potential of PENG, TENG, and the hybrid varieties of nanogenerators, a few challenges exist both in the nanogenerator functionalities and in the phase separation process for development, which in fact hinder the commercial scale-up of the products.

1. The phase-separated functional layer formation needs optimization for numerous conditions. Depending on the nature of phase separation, the composition of the solvent, temperature of processing, vapor exposure, time required, thickness of coating, etc., need accurate optimization. This can make the prerequisites lengthy.
2. PENG functionality typically depends on the dipole alignment happening in the molecular structure, which is often done by poling or electrospinning. However, for the phase separation process, this happens through the polarization of phases. This leaves the risk of non-uniformities in the dipole alignment, which can decrease the output performance.
3. Phase separation leaves porous structures that sometimes cause connecting channels for electron transfer. Depending on the particles used for reinforcement, the composite layers can form interconnected conducting networks, which is not feasible for the nanogenerator’s performance.
4. Durability issues can occur due to repeated mechanical vibrations, ultra sound, and the contact electrification process. The chances are enhanced for porous, thin, and mechanically unstable phase-separated structures. It is highly desirable to strengthen the individual layers through additional reinforcement strategies.
5. Crystallinity, thermal and mechanical properties, and structural significance of the composites are often addressed when studying the PENGs and TENGs. In addition to these physical properties, it is desirable to explore chemical functionalities that could be helpful in designing multi-functional applications.
6. A sustainable future depends on artificial intelligence, cloud computing, and robotics, and the PE and TE NGs are very optimistic about smart society 5.0. Therefore, theoretical studies and simulation (molecular dynamics, density functional theory, etc.) research are highly required for advanced applications.

While the TE/PE nanogenerators are developing, there is still plenty of scope for plenty of research and development, which could possibly lead to advanced and multi-functional soft materials. It can be envisioned that these materials will find exciting applications in a sustainable future society.
